# “Cell Disk” DNA Storage System Capable of Random Reading and Rewriting

**DOI:** 10.1002/advs.202305921

**Published:** 2024-02-08

**Authors:** Zhaohua Hou, Wei Qiang, Xiangxiang Wang, Xiaoxu Chen, Xin Hu, Xuye Han, Wenlu Shen, Bing Zhang, Peng Xing, Wenping Shi, Junbiao Dai, Xiaoluo Huang, Guanghou Zhao

**Affiliations:** ^1^ School of Ecology and Environment Northwestern Polytechnical University 1 Dongxiang Road, Chang'an District Xi'an Shaanxi 710129 P. R. China; ^2^ Shenzhen Key Laboratory of Synthetic Genomics Guangdong Provincial Key Laboratory of Synthetic Genomics Shenzhen Institute of Synthetic Biology Shenzhen Institutes of Advanced Technology Chinese Academy of Sciences Shenzhen Guangdong 518055 P. R. China; ^3^ Shenzhen Branch, Guangdong Laboratory of Lingnan Modern Agriculture, Genome Analysis Laboratory of the Ministry of Agriculture and Rural Affairs, Agricultural Genomics Institute at Shenzhen Chinese Academy of Agricultural Sciences Shenzhen P. R. China

**Keywords:** cell Disk, codec algorithm, DNA data storage, random reading and rewriting

## Abstract

DNA has emerged as an appealing material for information storage due to its great storage density and durability. Random reading and rewriting are essential tasks for practical large‐scale data storage. However, they are currently difficult to implement simultaneously in a single DNA‐based storage system, strongly limiting their practicability. Here, a “Cell Disk” storage system is presented, achieving high‐density in vivo DNA data storage that enables both random reading and rewriting. In this system, each yeast cell is used as a chamber to store information, similar to a “disk block” but with the ability to self‐replicate. Specifically, each genome of yeast cell has a customized CRISPR/Cas9‐based “lock‐and‐key” module inserted, which allows selective retrieval, erasure, or rewriting of the targeted cell “block” from a pool of cells (“disk”). Additionally, a codec algorithm with lossless compression ability is developed to improve the information density of each cell “block”. As a proof of concept, target‐specific reading and rewriting of the compressed data from a mimic cell “disk” comprising up to 10^5^ “blocks” are demonstrated and achieve high specificity and reliability. The “Cell Disk” system described here concurrently supports random reading and rewriting, and it should have great scalability for practical data storage use.

## Graphical abstract

A novel “Cell Disk” DNA storage system, which engineers yeast cells as addressable chambers to store high‐density DNA‐encoded information, concurrently supports random reading and rewriting, making it promising for a variety of large‐scale data storage applications.

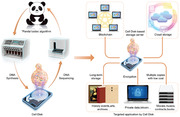

## 1 Introduction

**Figure 1:**
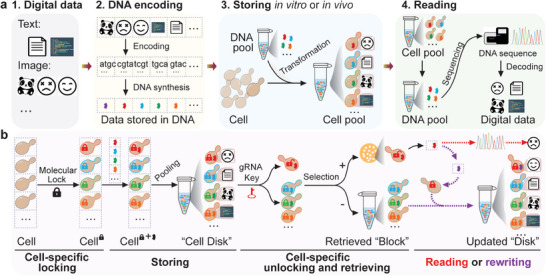
Schematic illustration of the generic or “Cell Disk” DNA data storage. a) The generic framework for DNA‐based storage includes encoding of digital data to synthetic DNA sequences(encoding), storing of DNA in a pool either in vitro or in vivo (storing), sequencing of DNA, and decoding the information (reading). b) The “Cell disk” allows for both random reading and re‐rewriting. In the system, cells are barcoded by unique molecular “locks” that can be unlocked by the corresponding gRNA “keys”. These cells are employed as distinct “disk blocks” for archiving different synthetic DNAs represented by the line segments marked by various colors, and pooled together to form a “Cell Disk”. Each “cell block” can be unlocked by introducing the corresponding gRNA “key” and followed by selection (“+”) or counter‐selection (“‐”) to achieve targeted retrieval or erasure. Data stored in these “cell blocks” can be read and/or rewritten, and subsequently returned to the “Cell Disk” for data update or expansion.

DNA provides an attractive medium for dense and long‐lasting data storage, which is desperately needed due to explosive data growth.^[^ ^1^ ^]^ A routine DNA data storage system performs four main steps: digital data are encoded into a series of synthetic DNA sequences, stored in vitro or in vivo as a pool, that are read by DNA sequencing and decoded back into digital information (**Figure** **1**a). In recent years, much effort has been placed on implementing these steps, and the concept of DNA data storage has been moving from a “prototype idea” to “practical application”.^[^ ^1^ ^]^ Fundamental data storage functions, such as repeatedly accessing the data files of interest and arbitrarily operating on the in‐storage data files, are therefore sorely needed for DNA‐based data storage.

While it is generally impractical to retrieve and update the desired data items by sequencing and/or rewriting all the DNA in a pool due to performance and cost considerations, various innovative approaches have been devised for selective retrieval of data from a pool of DNA‐encoded files in the past few years. Initially, polymerase chain reaction (PCR) was employed to selectively access data by amplifying the target DNA sequences from a DNA pool using primers corresponding to the random‐access adaptors on the sequences.^[^ ^2^ ^]^ Earlier work even successfully achieved data retrieval from DNA pools storing 200 MB data using this method.^[^ ^3^ ^]^ Nevertheless, substantial inherent drawbacks have been found, including the high data storage space cost for encoding random‐access adaptors, irreversible consumption or damage of the DNA pool by amplification, and the considerable difficulty of amplifying large DNA‐stored data files (PCR can generally amplify data sizes below 10 kb).^[^ ^1^, ^4^ ^]^ Recently, several novel methods were developed for randomly accessing DNA‐stored data, e.g., replacing PCR amplification by magnetic bead extraction using biotin‐labeled primers or using surface‐labeled single‐strand DNA barcodes to access individual physically segregated data files in silica capsules.^[^ ^4^ ^]^ However, these methods still possess considerable limitations with regard to data reusability, experimental complexity, and tradeoffs in reusability, storage density or scalability. Moreover, current DNA‐based storage systems generally lack data rewriting capability, limiting their applications to read‐only data storage.^[^ ^1^, ^5^ ^]^ Inspired by the inherent ability of cellular reproduction to replicate information, researchers have engineered various cell genetic systems for data storage that open the door for in vivo DNA data storage.^[^ ^6^ ^]^ One recent study described an inner‐cell genome editing method that successfully rewrote a few “words” from DNA‐stored data, yet it may be challenging to manipulate large data files by this method, and it does not take random reading of data into account.^[^ ^5^ ^]^ On the whole, the present DNA data storage systems typically have difficulty randomly reading or rewriting large data files and generally do not achieve random reading and rewriting in a single system, severely restricting their practical applications.^[^ ^1b,c^ ^]^

Here, we develop a DNA storage system capable of random reading and rewriting, referred to as a “Cell Disk” (Figure 1b). In this system, yeast cells are first barcoded by unique CRISPR/Cas9‐based “locks” that contain constitutively inactive marker genes whose expression can be switched on by the corresponding gRNA “keys”. These locked cells are employed as distinct “disk blocks” for archiving different DNA‐encoded information and are then pooled together to form a “Cell Disk”. Based on this data storage architecture, targeted retrieval or erasure of the desired “cell block” from up to 10^5^ “blocks” has been achieved through selection or counterselection upon request after introducing the corresponding gRNA “key”. Subsequently, the data stored in the chosen “cell block” can be easily read by sequencing and/or rewritten and then returned to the “Cell Disk” for data update or expansion. Moreover, a codec algorithm with lossless compression that controls bio‐constraints well is also developed to achieve high‐density in vivo data storage. Collectively, the “Cell Disk” system achieves random data retrieval and renewal and increases data density, which paves the way for future industrially scalable DNA data storage.

## 2 Results

### 2.1 CRISPR/Cas9‐Based “Lock‐And‐Key” for Targeted Retrieval of the Desired Cell Type from up to 10^5^ Distinct Cell Types

**Figure 2:**
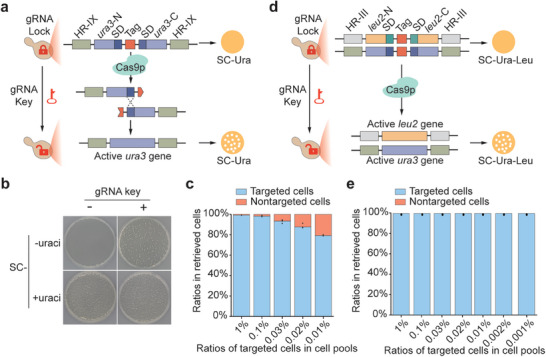
Design and optimization of the CRISPR/Cas9‐based “lock‐and‐key” modules enable selective and precise retrieval of the desired cell type from a scalable cell pool. a) Working model for CRISPR/Cas9‐based “lock” and key”. In the lock module, the inactive *ura3* marker gene with segmental duplication (SD) is divided into two parts (*ura3‐*N and *ura3‐*C) by the insertion of a unique gRNA target sequence (Tag) and two flanking homologous arm sequences (HRs) (Figure S1a, Supporting Information), used for inserting the “lock” module into the yeast genome to barcode (or lock) the cell. Once the locked cell is introduced with the corresponding gRNA key (Figure S1b, Supporting Information), Cas9 will be guided to cleave the gRNA target sequence to generate double‐strand break, which will be repaired through homologous recombination between the SD sequences, resulting into the restoration of the active full‐length *ura3* gene to generate the unlocked cells. Therefore, the unlocked cells but not the locked cells can grow on the synthetic medium without uraci (SC‐Ura), allowing for selective retrieval of the desired cell. b) The locked cells are transformed with either a plasmid expressing the corresponding gRNA key (+) or an empty plasmid (‐), and plated onto the synthetic medium with (+) or without (‐) uraci. c) Relative ratios of targeted and nontargeted cells obtained on SC‐Ura medium after transformation of the gRNA key plasmid into various cell pools comprising different ratios (ranging from 10^2^:1 to 10^4^:1) of these two types of locked strains. d) Working model for the double‐checked “lock”. In this design, each cell is locked by two lock cassettes harboring dual selection marker genes (*ura3* and *leu2*), and when transformed with the gRNA key, only the cells with both marker genes restored can grow on the synthetic medium without uraci and leucine, rendering the specificity to be double‐checked. e) Relative ratios of targeted and nontargeted cells obtained on SC‐Ura‐Leu medium after transformation of the gRNA key plasmid into various cell pools comprising different ratios (ranging from 10^2^:1 to 10^5^:1) of these two types of strains harboring the double‐checked locks as shown in d).

CRISPR‐associated protein 9 (Cas9) is a well‐known enzyme that can be programmed to cut a target DNA locus guided by the corresponding guide RNA (gRNA).^[^ ^7^ ^]^ Based on the CRISPR/Cas9 system, a “lock‐and‐key” module was developed to achieve controllable expression of the selection marker gene, as shown in **Figure** **2**a. The “lock” module contains an inactive marker gene with segmental duplication (SD) divided by the insertion of a unique gRNA target sequence (Tag) and two flanking homologous arm sequences (HRs) (Figure S1a, Supporting Information), which is used for inserting the “lock” module into the yeast genome to barcode (or lock) the cell. Once the locked cell is transformed with the gRNA “key” (Figure S1b, Supporting Information), Cas9 will be guided to cleave the target sequence and generate a double‐strand break, which will be repaired by the homologous recombination machinery, leading to the expression of an active marker gene.

As shown in Figure 2b, many transformants grow on the synthetic medium without uraci (SC‐Ura) after introducing the gRNA into a “locked” yeast strain that harbors an inactive *ura3* marker gene and therefore prevents growth on the SC‐Ura medium, suggesting that the gRNA key can efficiently switch on *ura3* expression. Following this idea, we set out to see if this approach could enable targeted retrieval of a desired cell type from a population of diverse cell types. As shown in Figure S2a (Supporting Information), two types of locked strains equipped with different gRNA targets were pooled together in different ratios (ranging from 100:1 to 10 000:1) to determine the sensitivity of this approach. To facilitate monitoring, the *ade2* gene of the nontargeted cells was eliminated, allowing these cells to turn red in synthetic medium supplied with a low dosage of adenine (SC‐Ade^low^). The pooled yeasts were then transformed with the gRNA “key” and plated onto the SC‐Ura‐Ade^low^ medium. The results showed that the desired white‐colored cells were obtained in all groups (Figure S2b, Supporting Information). However, nontargeted red‐colored cells also appeared in all groups, although in low numbers (Figure S2b, Supporting Information), which might be attributed to an unintended low‐rate recombination between the SD sequences within the “lock” cassette.^[^ ^8^ ^]^ Their ratio rose as the ratio of the targeted cells in the original pool fell (Figure 2c), compromising the accuracy and scalability of this approach for selective data retrieval. We argue that inserting an extra copy of the lock cassette equipped with another *leu2* marker gene (Figure 2d) will reduce the probability of restoring two marker genes exponentially considering the low spontaneous recombination frequencies in the yeast *Saccharomyces cerevisiae* (Figure S2b, Supporting Information).^[^ ^9^ ^]^ As expected, when the cells of interest were double‐checked, they were successfully retrieved with 100% accuracy without discernible off‐targeting effects, and the accuracy remained constant when one target cell type was buried in up to 10^5^ different cell types (Figure 2e; Figure S2c,d, Supporting Information). This “lock‐and‐key” approach demonstrates high specificity and robust reliability with a selection sensitivity of one in 10^5^ cells.

### 2.2 Panda Codec Algorithm with Lossless Compression to Increase the Information Density

**Figure 3:**
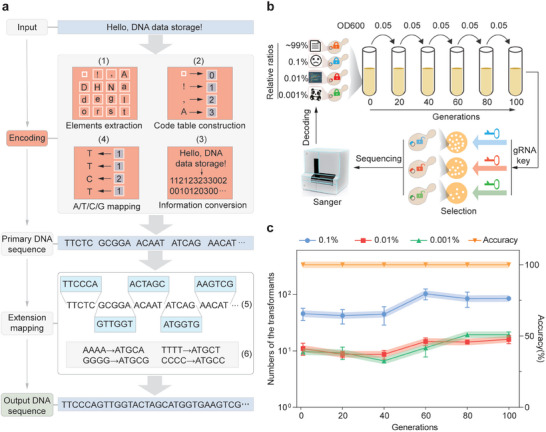
Encoding and random reading of compressed data in a “Cell Disk”. a) The pipeline of “Panda” algorithm. It can be divided into five main sections: data input, encoding, primary DNA sequence conversion, extension mapping, and DNA sequence output. To encode the information, character compositions from text or digital number (0, 1, 2…9) compositions of pixel values from images are extracted and taken as “element sequence” (1). This sequence is first used to generate a code table (2) and then a compression mapping process is employed to encode the data into decimal values, which are then converted into quaternary numbers (0, 1, 2, 3) (3) corresponding to A/T/C/G sequences (4). The acquired base sequence is separated into 5‐base units and subjected to extension mapping to GC contents and homopolymer repeats (5). Further, “AAAA”, “TTTT”, “GGGG”, and “CCCC” are replaced for the maintain homopolymer runs below 4 nt (6). b) Schematic of recurrent data retrievals from the“Cell Disk”. Four types of cells, each storing unique information, were mixed at the indicated ratios to create a yeast pool referred to as a “Cell Disk”. During a passaging experiment lasting 100 generations, the yeast pool was repetitively cultured for 20 generations (OD600 from 0.05 to 2) and the subcultures from every 20 generations were harvested and transformed with a set of gRNA keys for retrieving specific datasets. The accuracy of the retrieved data was further evaluated by PCR using primers targeting data‐encoded DNA sequences (Table S2, Supporting Information) and DNA sequencing. c) Numbers of the transformants and the accuracy of the retrieved data obtained by introducing particular gRNA keys into the subcultures from various generations were shown.

To achieve practical data storage, we developed a codec algorithm, named “Panda”, for converting data into DNA sequences and vice versa. The essential logic of classical LZW compression was integrated into this algorithm to store as much data as possible. Moreover, to simplify the data codec process, we process the data by reducing the binary conversion steps (**Figure** **3**a). First, character compositions from text or digital number (0, 1, 2…9) compositions of pixel values from images were extracted and taken as the “element sequences”. Each sequence was first used to generate a code table, and then a compression mapping process was employed to encode the data into decimal values, which were then converted into quaternary numbers (0, 1, 2, 3) corresponding to A/T/C/G sequences (Note S1 and Figure S3, Supporting Information). To control the bio‐constraints, including GC content and homopolymer runs in the sequence, a two‐step extension mapping process as described in our recent work was employed.^[^ ^10^ ^]^ In the first extension mapping step, each five‐nucleotide unit was mapped to a six‐nucleotide unit with the GC content strictly controlled at 50%. Then, the homopolymer repeats “AAAA”, “TTTT”, “GGGG”, and “CCCC” was replaced by “ATGCA”, “ATGCT”, “ATGCG” and “ATGCC”, respectively, at the second extension mapping step (Figure S4, Supporting Information). By doing this, we strictly controlled the GC content of the encoded sequence to ≈50% and the homopolymer runs to below 4 nucleotides at both the whole and regional sequence levels (Figure S5 and Table S1, Supporting Information). This should enable Panda to be very suitable for encoding data into the long genes used for in vivo data storage, which generally employ a gene assembly process that requires GC content even at the regional level (e.g., 100 bp). Given that Panda integrates a classical LZW compression logic, it shows a higher information density (information capacity per nucleotide) than many current developed DNA data storage algorithms,^[^ ^3^, ^6^, ^11^ ^]^ with 3.39 bits/nt on average for the five stored data files (Note S1 and Table S1, Supporting Information). This also yields the highest physical density of DNA data storage, 770.8 Eb/g (Figure S9, Supporting Information). Since LZW has been widely used in lossless compression for silicon‐based data storage, it is believed that the “Panda” algorithm should make our “Cell Disk” system very useful for practical data storage with high density (Note S1, Supporting Information).

### 2.3 Random Reading of Compressed Data from the “Cell Disk”

Yeast cells have been shown to be able to be used as a chamber, similar to a “block” in a hard disk, to store over two hundred kilobases of DNA‐encoded data,^[^ ^6a^ ^]^ which can be pooled together to generate a “Cell Disk”. Following this idea, to test the “lock‐and‐key” approach for random access of practical DNA‐encoded data in a “Cell Disk”, we encoded 4 different samples of digital data into four 3000‐base pair DNA sequences with the Panda algorithm and further synthesized and cloned these DNA sequences into *E. coli*‐yeast shuttle plasmid vectors (Figure S1c, Table S2, and Note S2, Supporting Information), which were then transformed into yeast cells to store the four samples of data in “cell blocks”. As shown in Figure 3b, the 4 types of “cell blocks” were pooled together to form a “Cell Disk”, with each “block” occupying a distinct ratio of the disk. Next, we investigated whether the desired subsets of data could be selectively retrieved. Different gRNA “keys” were individually transformed into the cell pool to capture the target cell “block”. A great number of colonies were then obtained from the selection medium within each group (Figure S6a, Supporting Information, “Generation 0”), suggesting the high robustness of our method. To further verify the fidelity of the retrieved data, transformed colonies were then randomly chosen from each experimental group and verified by PCR using primers targeting data‐encoded DNA sequences. It was shown that arbitrary subsets of data, occupying as low as 0.001% of the “Cell Disk”, could be selectively retrieved with 100% accuracy (Figure 3c; Figure S6b, Supporting Information, “Generation 0”).

Given that a practical data storage system generally requires repeatable data access, we further set out to evaluate the potential of this cell‐based self‐replicative storage system for continuous data access. We then devised a cell passaging experiment (Figure 3b) in which the cell pool was repeatedly cultured for 20 generations (OD600 of 0.05 to 2) in fresh media before harvesting for the next culturing cycle. During a 100‐generation passaging experiment, cells from every 20 generations were employed to assess the stability of random reading for long‐term data storage. Our results show that after 100 generations of automatic database regeneration, the desired information could still be selectively and precisely retrieved with no discernible loss of accuracy or robustness (Figure 3c), which is validated by PCR using data‐specific primers and Sanger sequencing (Figures S6b and S7, Supporting Information, Supporting Information). In summary, these results show that the “Cell Disk” storage system is a robust platform that enables random and repeatable data access without detectable off‐target effect.

### 2.4 Random Rewriting of the Compressed Data from the “Cell Disk”

**Figure 4:**
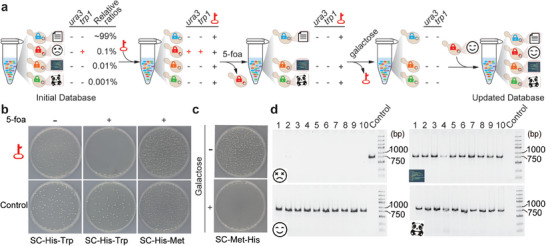
Random rewriting of the compressed data from the “Cell Disk”. a) Schematic of the process for random rewriting of information in the “Cell Disk”. In the initial “Cell Disk”, all of these cells were barcoded by unique molecular locks harboring inactive *ura3* genes. In addition, the *trp1* gene of the nontargeted cells was eliminated, which prevents their growth on synthetic medium without tryptophan (SC‐Trp), allowing targeted and nontargeted cells to be discriminated simply by colony formation. When the gRNA “key” targeting for the cells carrying the “cry” image was introduced into the cell pool, the targeted cells acquired the complete *ura3* gene and could be selectively removed from the cell pool after 5‐foa treatment. To recycle the cell pool for future usage, a galactose‐inducible gRNA expression cassette was preinstalled in the gRNA key expression plasmid (Figure S8, Supporting Information) to express a gRNA that guides Cas9 to cleave and eradicate the plasmid efficiently upon galactose induction. Finally, the recycled cell pool, with the “cry” image removed, was expanded by adding another cell “block” storing a “smile” image, demonstrating targeted rewriting of specific data to achieve data update. b) Colony formation analysis of pooled cells that were transformed with either a plasmid expressing the gRNA key or an empty plasmid, and plated onto the synthetic medium with (+) or without (‐) 5‐foa. c) After galactose induction, the gRNA key expression plasmid was effectively removed from the cell pool. d) PCR validation of the data from the updated database using primers targeting data‐encoded DNA sequences (Table S2, Supporting Information).

While current approaches allow for random data access, a practical DNA‐based storage system would require not only reading but also completely rewriting arbitrary data within the dataset to update it.^[^ ^1^, ^5^ ^]^ This is currently a very difficult task since it is generally difficult to extract the targeted DNA data file completely.^[^ ^2^, ^4^, ^5^ ^]^ Inspired by the counterselection operation used in molecular biology to eradicate the cells harboring a selection marker gene, we attempted to introduce this method into our system to achieve complete erasure of the cell carrying the desired data from a DNA‐stored dataset. First, a mimic DNA‐stored dataset comprised of pooled cells was constructed (**Figure** **4**a). Within it, the *trp1* gene of the nontargeted cells was eliminated, which prevents their growth on synthetic medium without tryptophan (SC‐Trp), allowing counterselection to be evaluated simply by colony formation. To specifically switch on the expression of the *ura3* marker gene in the target cells carrying the “cry” image, the cell pool was transformed with the corresponding gRNA key. The transformants were then counterselected in medium containing 5‐fluoroorotic acid (5‐foa), a chemical that can be catalyzed into a toxin by URA3p.^[^ ^12^ ^]^ After counterselection, the cell mixture was plated onto SC‐Trp medium for the colony formation assay. As shown in Figure 4b, there were no colonies growing on the SC‐Trp medium when the cells were transformed with the gRNA key and counter‐selected, while there were many colonies growing on the SC‐Trp medium without 5‐foa counterselection and/or gRNA transformation, suggesting that counterselection can be used to completely remove particular cells along with their cargo data from the cell pool. In addition, to recycle the cell pool for future usage, our system has a simple way to eliminate the plasmid expressing the gRNA key. A galactose‐inducible gRNA expression cassette was preinstalled in the gRNA key expression plasmid (Figure S8, Supporting Information) to express a gRNA that guides Cas9 to cleave and eradicate the plasmid efficiently upon galactose induction, as shown in Figure 4c and our previous study.^[^ ^13^ ^]^ To further demonstrate the entire cycle for rewriting specific data to update the DNA‐encoded datasets, the recycled cell pool, with the “cry” image removed, was expanded by adding another cell “block” harboring the synthetic DNA coding for a “smile” image, which employed the same gRNA key as was previously used for querying the “cry” image in the “Cell Disk”. Finally, when different gRNA keys were used to extract the target data from the updated dataset, the results revealed that all of the data were successfully retrieved and that the “cry” image in the original Cell Disk was rewritten as the “smile” image in the updated Cell Disk (Figure 4d). Together, these results show that the “Cell Disk” storage system enables targeted rewriting of specific data to update the stored information.

## 3 Discussion

DNA‐based data storage is an attractive alternative to today's popular silicon‐based storage solutions.^[^ ^1^ ^]^ By 2027, it is predicted that the global market for DNA data storage will be worth ≈$1.5 billion.^[^ ^14^ ^]^ The use of DNA as a data storage medium is becoming more realistic. However, the present DNA data storage technology, which aims to store large amounts of data, struggles to perform random reading and writing in a single system, making it challenging to scale for useful big data storage.^[^ ^1b,c^ ^]^ In particular, for data storage over 10 Kb, which often requires a pool of synthesized DNA sequences or in vivo cells to store the data, rewriting one specific bit of data might be required to resynthesize the entire DNA pool or make all the in vivo cells, resulting in a significant cost waste.^[^ ^1c^ ^]^ Here, we provide a proof‐of‐concept framework for a scalable DNA‐based “Cell Disk” storage system capable of random reading and rewriting to address these difficulties. This system allows for the selective extraction and erasure of desired files from a pool of up to 10^5^ different data files, enabling targeted data retrieval and/or update by sequencing or resynthesizing only the selected DNA and not the entire DNA pool, enabling scalable DNA data storage.

**Figure 5:**
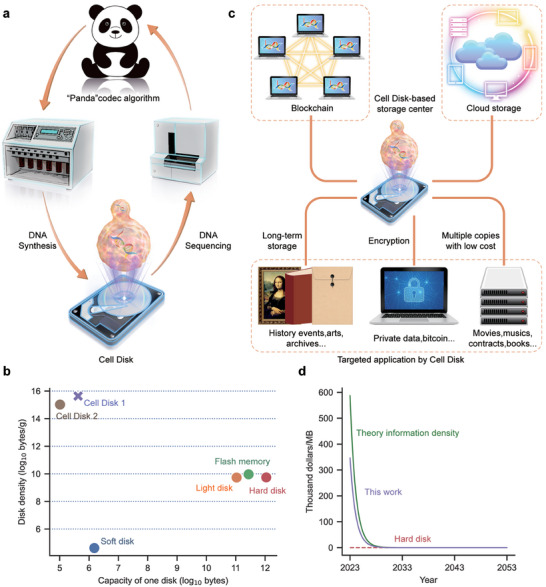
Illustration of “Cell Disk” applications. a) The complete “Cell Disk” storage system. It involves four parts: “Panda” codec algorithm, DNA synthesis, DNA sequencing, and “Cell Disk”. b) Disk densities comparison among different disks. Cell Disk 1, the DNA used to store information in one cell is proposed to be 1 MB; Cell Disk 2, the DNA used to store information in one cell is proposed to be 242 kB as shown by Yuan et al.^[^ ^6a^ ^]^ The density of these “Cell Disks” exceeds other storage mediums by at least four orders of magnitude. c) The application of “Cell Disk”. The “Cell Disk” storage method has significant advantages in data storage, such as long‐term storage, encryption capability, and low‐cost storing of numerous copies of cold data. Furthermore, it can support specific data storage applications such as blockchains and cloud storage based on DNA. d) Cost projection for Cell Disk. Supposing the cost of DNA synthesis would decrease following Moore's Law, the cost of “Cell Disk” is projected. The red dashed line represents the cost projection of hard disks; the green solid line represents the cost projection of DNA data storage with a density of 2 bits/nt. Approximately 50 years from now, the cost of DNA data storage will approach that of hard disks. The purple solid line displays the cost projection of DNA data storage by “Cell Disk”.

The “Cell Disk” system operates by encoding, synthesizing, and archiving DNA into individual cell chambers in a parallel manner and can be easily scaled up to store massive amounts of data. A highly tractable and manipulable cell‐based physical storage structure was implemented in a “Cell Disk” to organize hundreds of thousands of individually addressable cell chambers using an innovative molecular “lock‐and‐key” module coupled with selection or counterselection operations. This system is a novel method of targeted data retrieval, which circumvents several lingering drawbacks of the predominant PCR‐based method and its derivatives. Its advantages include 1) eliminating the need to sacrifice valuable storage space for encoding indices and PCR adaptors, which normally accounts for over 1/4th of the oligo‐DNA length in a synthesized oligo‐pool;^[^ ^3^, ^4^ ^]^ 2) overcoming the difficulty of amplifying large DNA fragments (one gRNA (20 nt) in our system can query one cell chamber capable of storing megabase‐length exogenous DNA,^[^ ^15^ ^]^ whereas PCR can only amplify DNA tens to thousands of bases in length); 3) eliminating the physical linkage between the index and payload sequences, allowing the index and payload sequences to be synthesized and rewritten separately to implement a flexible storage architecture; and 4) enabling highly repeatable data access by engineering cell replication for data self‐regeneration. Notably, this system implements a random rewriting capability, which distinguishes it from previous systems and makes it extremely useful for updating specific datasets without having to synthesize the entire DNA pool. Additionally, the Panda codec algorithm, which combines the fundamental logic of LZW compression, bio‐constraint control, and data conversion, was developed to computationally write and read the “Cell Disk”, achieving a quite high information density of 3.39 bits/nt for tested practical data storage. Similar to other work that incorporates analogous data conversion or compression logic,^[^ ^16^ ^]^ this density can be further improved for the storage of more practical data applications (Figure S9 and Tables S1 and S4, Supporting Information). By connecting DNA synthesis and DNA sequencing, our work constitutes a full “Cell Disk”‐based DNA data storage system (**Figure** **5**a). The capacity of the disk is determined by the storage capacity of each cell as well as the selective specificity of random reading and writing. While yeast has been demonstrated to store over megabase‐length exogenous DNA per cell,^[^ ^15^ ^]^ based on our method, each Cell Disk may potentially store 39.456 GB of information given its targeted specificity in random writing and reading from a mimicked disk containing 10^5^ cell blocks. Although its capacity is smaller than that of other disks, its storage density is more than 10^4^ times greater than those of currently available soft disks, light disks, flash memory, and hard disks (Figure 5b). It appears to be quite promising for large‐scale data storage, although highly dense data storage may be needed. Moreover, owing to the benefits of in vivo DNA data storage, our “Cell Disk” system should at least be beneficial for the long‐term storage of data related to historical events or archives, high‐privacy data such as bitcoin pins, and data that can be stored as multiple copies at a low cost, such as music, movies, commercial contracts, and books. Additionally, it should encourage the construction of blockchain data centers and cloud storage facilities based on DNA (Figure 5c). Overall, our system should be appropriate for a variety of data storage scenarios.

Since the “Cell Disk” is a proof‐of‐concept technology, numerous major difficulties must be addressed before practical implementation. For example, the upper limit of artificial DNA acceptable by the host cell and the biological impacts of different data‐stored DNA sequences, especially their long‐term dynamics when stored in populated cells, are important in terms of capacity and stability and need to be clarified. In addition, although data‐stored DNA sequences were naturally copied with high fidelity during cellular replication, a flexible coding scheme being capable of error correction could be further explored to enhance the robustness of this system.^[^ ^6a^ ^]^ Another major problem is that the current cost of Cell Disks is not inexpensive. The current cost of DNA synthesis is quite high, making the cost of the whole “Cell Disk” orders of magnitude greater than those of other existing disks. As the storage system size is increased for large‐scale data storage, the cost of personnel and reagents in the experimental procedures also rises dramatically. These could have an impact on current disk applications. Nonetheless, as biochemical experiments develop in future years, such costs may soon decline. As novel DNA synthesis technology emerges, such as enzymatic DNA synthesis and chip‐based DNA synthesis, the cost of DNA synthesis will fall over the years.^[^ ^17^ ^]^ Moreover, automation and miniaturization of these procedures including engineering yeast cells, writing and/or reading DNA, despite still in fancy and to be integrated, have been realized respectively by the laboratory robots and/or microfluidics‐based techniques to reduce the process cost and time.^[^ ^18^ ^]^ These factors may enable the “Cell Disk” to progress much faster than current available disks, including flash memory, soft disks, light disks, and hard disks. While the first “hard disk” created by IBM in 1956 was a meter wide and high, held only 5 MB of data, and cost $50000 per MB,^[^ ^19^ ^]^ the current commercial “hard disk” is approximately the width of a palm, holds over 1 TB of data and costs only ≈$50. It is estimated that the price of a “Cell Disk” might reach the same level as that of a hard disk in ≈50 years if the cost decrease of DNA synthesis follows Moore's law (Figure 5d). At that time, the “Cell Disk” should have broad application prospects. In summary, the “Cell Disk” presented here should serve as a key technology with great potential for scaling up, and we hope that new improvements will make it a technological advancement that is industrially scalable.

## 4 Experimental Section

### Strains and Culture Conditions

The strains employed in this study are documented in Table S3 (Supporting Information). All yeast strains were cultivated at 30 °C using the following medium composition. The YPD medium contained 20 g L^−1^ glucose, 20 g L^−1^ tryptone, and 10 g L^−1^ yeast extract. For the Synthetic dropout (SC) medium, 6.7 g L^−1^ yeast nitrogen base and 20 g L^−1^ glucose were utilized, with the addition of the necessary amino acids. The SG medium shared the same components as SC, except that it contained 2% galactose instead of glucose. To prepare the 5‐foa medium, 1 g of 5‐Fluoroorotic acid was dissolved in 1 L of SC medium. Solid media were prepared by incorporating 1.5% (w/v) agar.

### Plasmid Constructions

For the expression of Cas9, a codon‐optimized *Cas9* gene from *Streptococcus pyogenes*, fused with a simian virus 40 (*SV40*) nuclear localization sequence, was cloned into an integration cassette. This cassette was placed under the control of the strong glyceraldehyde‐3‐phosphate dehydrogenase (GAP) promoter, with a cytochrome C1 (*CYC1*) terminator.^[^ ^20^ ^]^ To construct the gRNA “key” plasmid, the targeting gRNAs were cloned into generic gRNA cassettes. These cassettes contained the hepatitis delta virus (HDV) ribozyme and structural RNAs, flanked by the small nucleolar RNA 52 (*SNR52*) polymerase III promoter and a Suppressor 4 (*SUP4*) terminator.^[^ ^21^ ^]^ Additionally, a structural RNA containing a 20‐bp target gRNA sequence, flanked by hammerhead (HH) and *HDV* ribozymes, was inserted under the control of the inducible pGalS promoter and a *CYC1* terminator.^[^ ^13^ ^]^ For the construction of the “lock” plasmid, the target gRNA with TGG was introduced into the *ura3* and *leu2* genes. A stop codon was added immediately after the target gRNA sequence to ensure premature interruption of translation. Furthermore, a 100 bp segment duplication (SD) was positioned on both sides of the target sequence to facilitate homology‐driven scarless repair after cleavage, forming the “lock” module. The *ura3* and *leu2* “lock” modules were inserted into Chromosome IX and III, respectively. The NAT and HygB genes were used as selectable markers. The primers and plasmids utilized in this study are listed in Table S2 (Supporting Information). The detailed DNA sequences of plasmids are listed in Note S2 (Supporting Information).

### Yeast Transformation

The experiment began by inoculating a fresh colony into 5 mL of suitable medium and incubating it overnight at 30 °C. The resulting culture was then transferred to 5 mL of fresh medium with an initial OD600 of 0.1 and grown until reaching an OD600 of 0.6–0.8. The cell pellet was collected, washed once with sterile ddH2O, and then washed with 500 µL of 0.1 m LiAc/TE solution. The pellet was resuspended in 100 µL of 0.1 m LiAc/TE solution. Next, 20 µL of the cell solution, along with the relevant DNA segments or plasmids, was added to 85.2 µL of a prepared transformation mixture. The transformation mixture consisted of 62.4 µL of PEG 3350 (50% w/v), 8.22 µL of 1 m LiAc, 9.58 µL of DMSO, and 5 µL of ssDNA. The mixture was thoroughly and gently mixed. The tubes were then incubated at 30 °C for 35 min, followed by incubation at 42 °C for 15 min. After the incubation, the cells were collected and cultured in 800 µL of appropriate liquid medium for an additional 2 h. Finally, the cells were collected and plated on the appropriate solid medium, and incubated at 30 °C for ≈3 days.

### Locked Cell Construction

The DNA segment carrying the “lock” module was obtained by digesting the plasmid with BsaI. The digested fragments were subsequently transformed into the ZHY277 strain. After being inoculated in YPD liquid medium at 30 °C for 2 h, the cells were harvested and plated on YPD solid medium supplemented with 100 mg L^−1^ of Nourseothricin sulfate and 100 mg L^−1^ of Hygromycin B. The resulting colonies were further confirmed by PCR.

### DNA Data Storage in Locked Cells

DNA information is encoded by the “Panda” algorithm and synthesized by BGI‐Gene(http://www.bgi‐write.com) (Note S2, Supporting Information). The DNA sequences were inserted into PRS413 plasmid, resulting in the formation of information plasmids (Figure S1c, Supporting Information). Subsequently, 100 ng of the information plasmids were transformed into the designated lock‐labeled cells and plated onto SC‐His agar plates. The colonies that grew and carried different information were selected and inoculated into 5 mL of SC‐His liquid medium, followed by overnight cultivation at 30 °C. The resulting cultures were mixed in various proportions to create a library of DNA information strains. The library was then stored at −80 °C for future use.

### Querying Compressed Data from “Cell Disk” using gRNA “Keys”

The yeast strains containing the datasets were cultured in SC‐His liquid medium at 30 °C overnight. To retrieve the desired dataset, 1 µg of the corresponding gRNA “key” was introduced into the cell pool, ten times of the gRNA “key” and the cell pool used for the desired dataset accounts for 0.001%. Subsequently, the cells were incubated in SC‐His‐Met liquid medium for 2 h and then plated onto SC‐His‐Met‐Ura‐Leu agar plates. Following incubation at 30 °C for ≈3 days, the information plasmid was verified through colony PCR. The yeast colony PCR procedure is as follows: First, an appropriate amount of cells were suspended in 30 µL of 0.05% NaOH solution and then heat at 98 °C for 25 min, followed by centrifugation at 2500 rpm for 10 s. Next, 5 µL of the supernatant was mixed with 1 µL of a 10 µm forward primer, 1 µL of a 10 µm reverse primer, 0.5 µL of Phanta Max Super‐Fidelity DNA Polymerase, 12.5 µL of 2×Phanta Max Buffer, 0.5 µL of dNTP Mix, and 4.5 µL of ddH_2_O. The thermocycling protocol consisted of the following steps: initial denaturation (95 °C, 3 min); followed by 28 cycles of denaturation (95 °C, 15 s), annealing (56 °C, 15 s), and extension (72 °C, 1 min 30 s); and final extension (72 °C, 10 min). To decode the DNA information, the information segment of the DNA was amplified by PCR using universal primers suitable for Sanger sequencing.

### Cell Passaging Assay

A cell passaging experiment was conducted to assess the stability of data access in the cell‐based self‐replicative storage system. The cell pool containing the datasets was cultured in SC‐His liquid medium at 30 °C overnight. The resulting culture was then diluted into 5 mL of fresh SC‐His liquid medium with an initial OD600 of 0.05 and grown until reaching an OD600 of 1.6. This period was considered as 5 passages. Re‐dilution and re‐cultivation were performed once every 5 passages and continued until reaching 100 passages. After each interval of 20 passages, the gRNA “keys” were individually introduced into the cell pool to query the desired data. The cells were subsequently recovered on SC‐His‐Met‐Ura‐Leu solid medium. The accuracy of data access was initially tested using colony PCR and further confirmed through Sanger sequencing, which are displayed in Figures S6b and S7 (Supporting Information).

### Random Rewriting of Dataset

A schematic diagram illustrating the process for dataset rewriting in this DNA storage system is presented in Figure 4, which involving three steps: 1) Transforming the match gRNA to activate the *ura3* gene in the targeting cells; 2) Eradiating the targeting cells from “Cell disk” by adding 5‐foa; 3) Updating “Cell disk” via adding the block with a new dataset. The details are described as follows: First, the cell pool containing the datasets was cultured in SC‐His liquid medium at 30 °C overnight. The gRNA “key” for rewriting the dataset was then introduced into the cell pool, followed by incubation in SC‐His‐Met liquid medium for 24 h. Second, the resulting culture was diluted into SC‐His+5‐foa liquid medium with an OD600 of 0.01 and incubated at 30 °C for 24 h. Thirdly, the culture was further diluted into SG‐His liquid medium with an OD600 of 0.01 and incubated at 30 °C for 60 h to remove the gRNA “key”. Finally, the plasmid containing the new dataset was transformed into the yeast strain carrying the same gRNA “key”, thereby achieving the rewriting of the dataset within the same strain.

## Conflict of Interest

The authors declare no conflict of interest.

## Author Contributions

Z.H., W.Q., and X.W. contributed equally to this work. Z.H., W.Q., X.W., J.D., X.H., and G.Z. developed the initial DNA data storage ideas. G.Z. and X.H. discussed and finalized the concept of “Cell Disk” system. Z.H. and X.W. designed and performed the biological experiments. X.C., X.H., X.H., W.S., B.Z., P.X., and W.S. provided significant intellectual input. W.Q. and X.H. designed and developed the codec methods. Z.H., X.W., and W.Q. prepared the initial manuscript draft. G.Z. and X.H. edited and finalized the manuscript. G.Z., X.H., and J.D. supervised and supported the study.

## Supporting information

Supporting Information

## Data Availability

The data that support the findings of this study are available in the supplementary material of this article.
