# Supplementary material for: “Cell Disk” DNA Storage System Capable of Random Reading and Rewriting
Source: Adv Sci (Weinh). 2024 Feb 8;11(15):2305921. doi: 10.1002/advs.202305921 (PMC11022697; doi:10.1002/advs.202305921)
Supplement: Supplementary file 1 — Supporting Information [file ADVS-11-2305921-s001.pdf]

ADVANCED SCIENCE
Open Access

Supporting Information

for *Adv. Sci.*, DOI 10.1002/advs.202305921

"Cell Disk" DNA Storage System Capable of Random Reading and Rewriting

Zhaohua Hou, Wei Qiang, Xiangxiang Wang, Xiaoxu Chen, Xin Hu, Xuye Han, Wenlu Shen, Bing Zhang, Peng Xing, Wenping Shi, Junbiao Dai, Xiaoluo Huang* and Guanghou Zhao**

Supporting Information

"Cell Disk" DNA storage system capable of random reading and rewriting

Zhaohua Hou, Wei Qiang, Xiangxiang Wang, Xiaoxu Chen, Xin Hu, Xuye Han, Wenlu Shen, Bing Zhang, Peng Xing, Wenping Shi, Junbiao Dai, Xiaoluo Huang*, and Guanghou Zhao**

Z. Hou, X. Wang, X. Chen, X. Hu, X. Han, W. Shen, B. Zhang, P. Xing, W. Shi, G. Zhao

School of Ecology and Environment

Northwestern Polytechnical University

1 Dongxiang Road, Chang'an District, Xi'an Shaanxi,710129, P.R.China

E-mail: zhaogh@nwpu.edu.cn

W. Qiang, X. Huang

1Shenzhen Key Laboratory of Synthetic Genomics, Guangdong Provincial Key Laboratory of Synthetic Genomics, Shenzhen Institute of Synthetic Biology, Shenzhen Institutes of Advanced Technology, Chinese Academy of Sciences, Shenzhen 518055, Guangdong, China

E-mail: huangxl@siat.ac.cn;

J. Dai

1 Shenzhen Branch, Guangdong Laboratory of Lingnan Modern Agriculture, Genome Analysis Laboratory of the Ministry of Agriculture and Rural Affairs, Agricultural Genomics Institute at Shenzhen, Chinese Academy of Agricultural Sciences, Shenzhen, China

2 Shenzhen Key Laboratory of Synthetic Genomics, Guangdong Provincial Key Laboratory of Synthetic Genomics, Shenzhen Institute of Synthetic Biology, Shenzhen Institutes of Advanced Technology, Chinese Academy of Sciences, Shenzhen 518055, Guangdong, China

E-mail: daijunbiao@caas.cn

Supplementary Figures

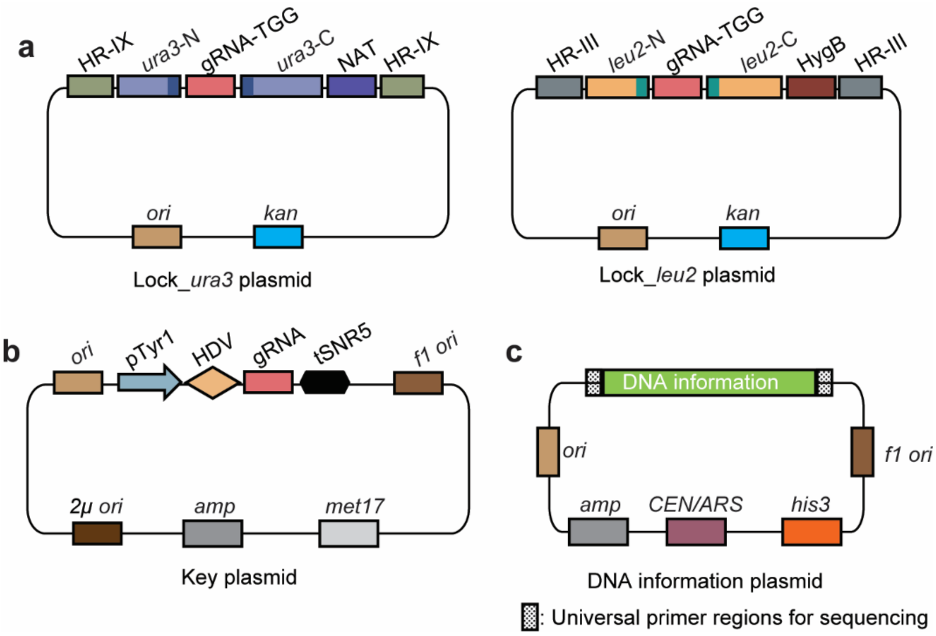

Figure S1. Schematic diagram of the "lock" a), "key" b) and DNA information plasmids c).

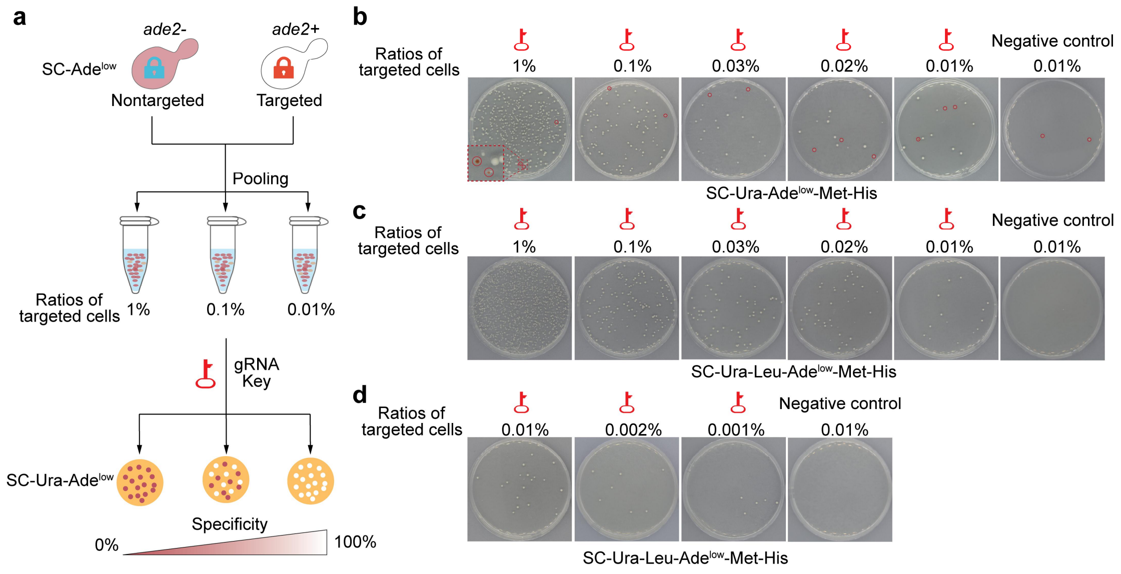

Figure S2. Retrieval of the desired cell type from a population of diverse cell types. a) Experimental design. The *ade2* gene of the nontargeted cells was eliminated to make these cells to turn red in synthetic medium supplied with a low dosage of adenine (SC-Ade^{low}), allowing for the discrimination of the targeted cells (white color) from nontargeted cells (red color). In this experiment, two types of locked strains including

targeted and nontargeted cells equipped with different gRNA targets were pooled together in different ratios as indicated. The pooled yeasts were then transformed with the gRNA "key", plated onto the selection medium and evaluated by the colors of the transformants to determine the specificity of this retrieval approach. b) Photographs of the transformation plates in retrieval experiments after introducing the plasmids with or without the gRNA key into the cell pool equipped with one lock (OD600 of 0.6 for each transformation experiment). The red colonies were labeled by the red circles and one representative part of the images (marked by the red rectangle) was enlarged to show the comparison between the red-colored and white-colored cells. c) and d) Photographs of the transformation plates in retrieval experiments after introducing the plasmids with or without the gRNA key into the cell pool equipped with dual locks (OD600 of 0.6 in C or 6 in D for each transformation experiment).

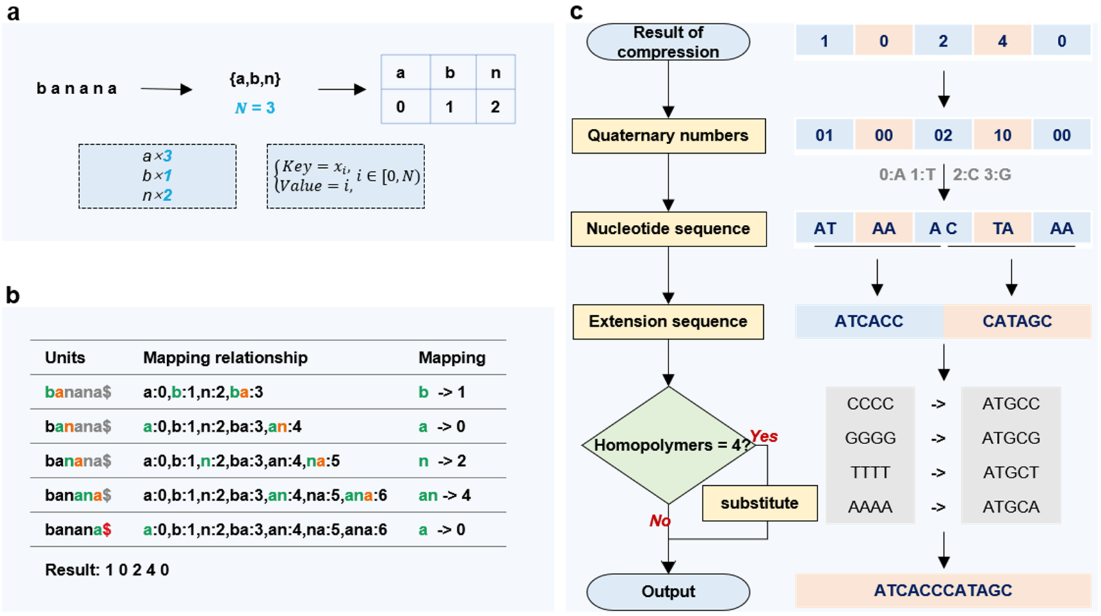

Figure S3. An example of "Panda" algorithm in encoding "banana" into DNA sequence. a) The initial code table construction. First, duplicates from the elements in the text are removed and (a, b, n) is obtained, which serves as the key for mapping. The initial code table is then created by assigning values to the mappings, started from "0". b) Data compression. Starting from the current position of the information to be

encoded, the longest key that matches an existing code in the code table is searched and its value is recorded. Afterwards, the matched key and the following element in the information to be encoded are taken as a new key and assigned with a new value, which expands the code table. c) Data encoding. The numerical list obtained from b) is firstly converted into a quaternary number. The number of digits for each quaternary number is determined by the number with the highest value in the list. The quaternary number is then encoded into a DNA sequence using the mapping relationship 0:A, 1:T, 2:C, 3:G. The resulting DNA sequence is then divided into 5 base units, which are expanded to 6 base units to manage the GC content in the sequence. Finally, we search the sequence for occurrences of CCCC/GGGG/TTTT/AAAA and replace them with ATGCC/ATGCG/ATGCT/ATGCA, limiting homopolymers to less than four.

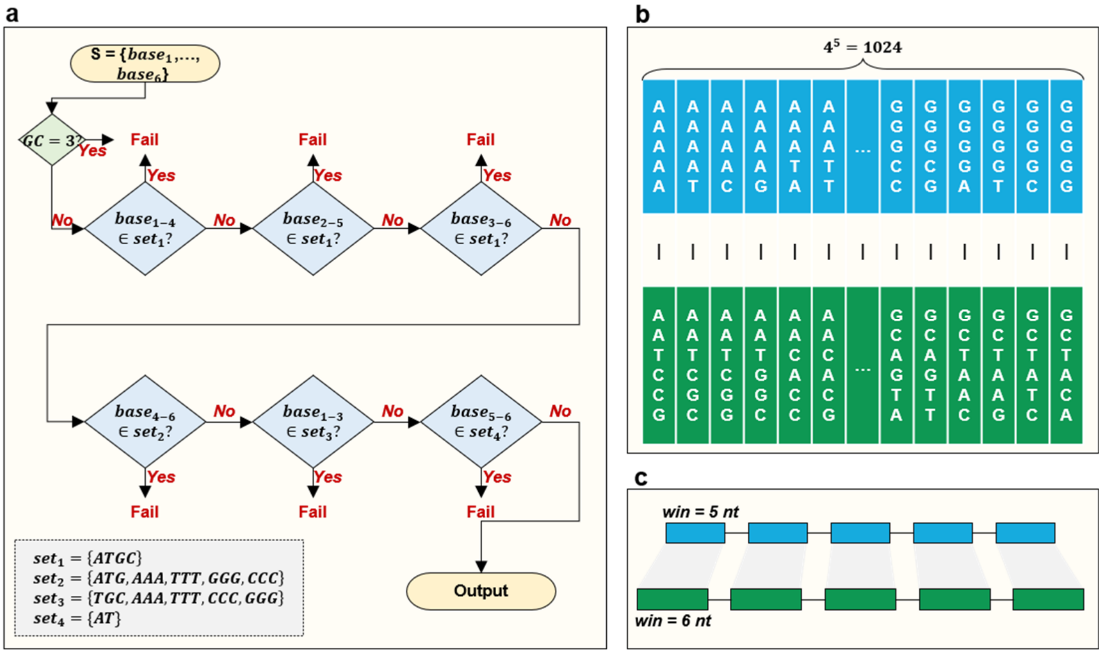

Figure S4. Illustration of the algorithm pipeline for GC controlling. a) 6-base units selection. To better control the GC content of the final encoded sequence and avoid the appearance of ATGCC/ATGCG/ATGCT/ATGCA sequences used for replacing four-base homopolymer runs in the sequence after extension mapping, corresponding filtering conditions are set. In the end, 1095 6-base units that meet the conditions are selected. b) Construction of the mapping table. A mapping code table from 5-base units

to 6-base units is constructed, where the 6-base units belong to the results received from step a). c) Sequence extension mapping. The original DNA sequence is divided into units of 5 bases, and the sequence units are extended based on the code table.

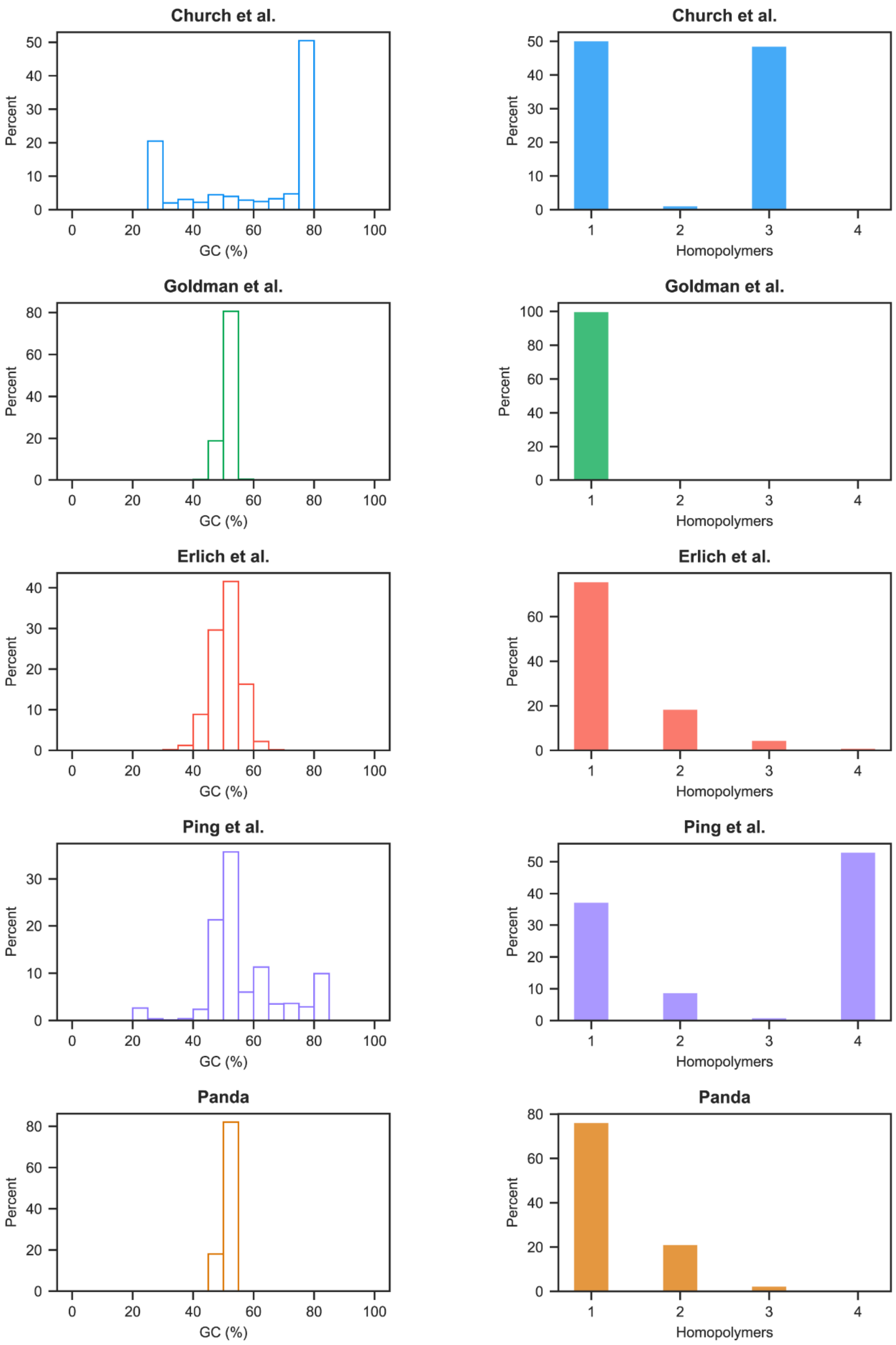

Figure S5. Sequence properties encoded by algorithms proposed by Church *et al.*,[1] Goldman *et al.*,[2] Erlich *et al.*,[3] Ping *et al.*,[4] and this work. The length of

Homopolymer runs and the regional GC content (window size = 100 nt) of sequences encoded by different algorithms are presented.

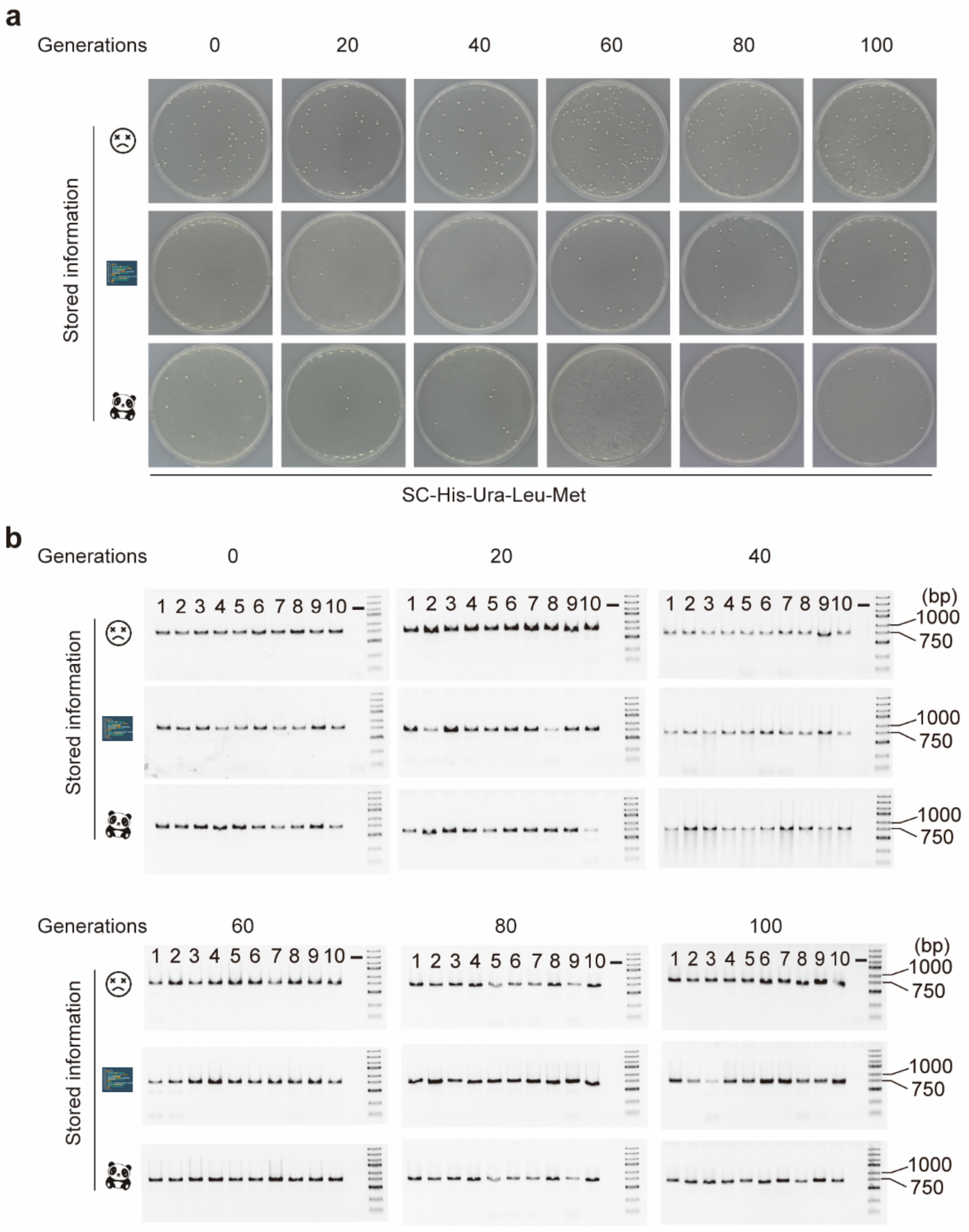

Figure S6. Results of iterative data access from "Cell Disk" by cell passaging experiment. a) The plate photographs of retrieval experiments through introducing particular gRNA keys into the subcultures from various generations. b) PCR analysis

of ten colonies randomly picked from the plates above to validate the presence of the retrieved data using primers targeting data-encoded DNA sequences (Table S2).

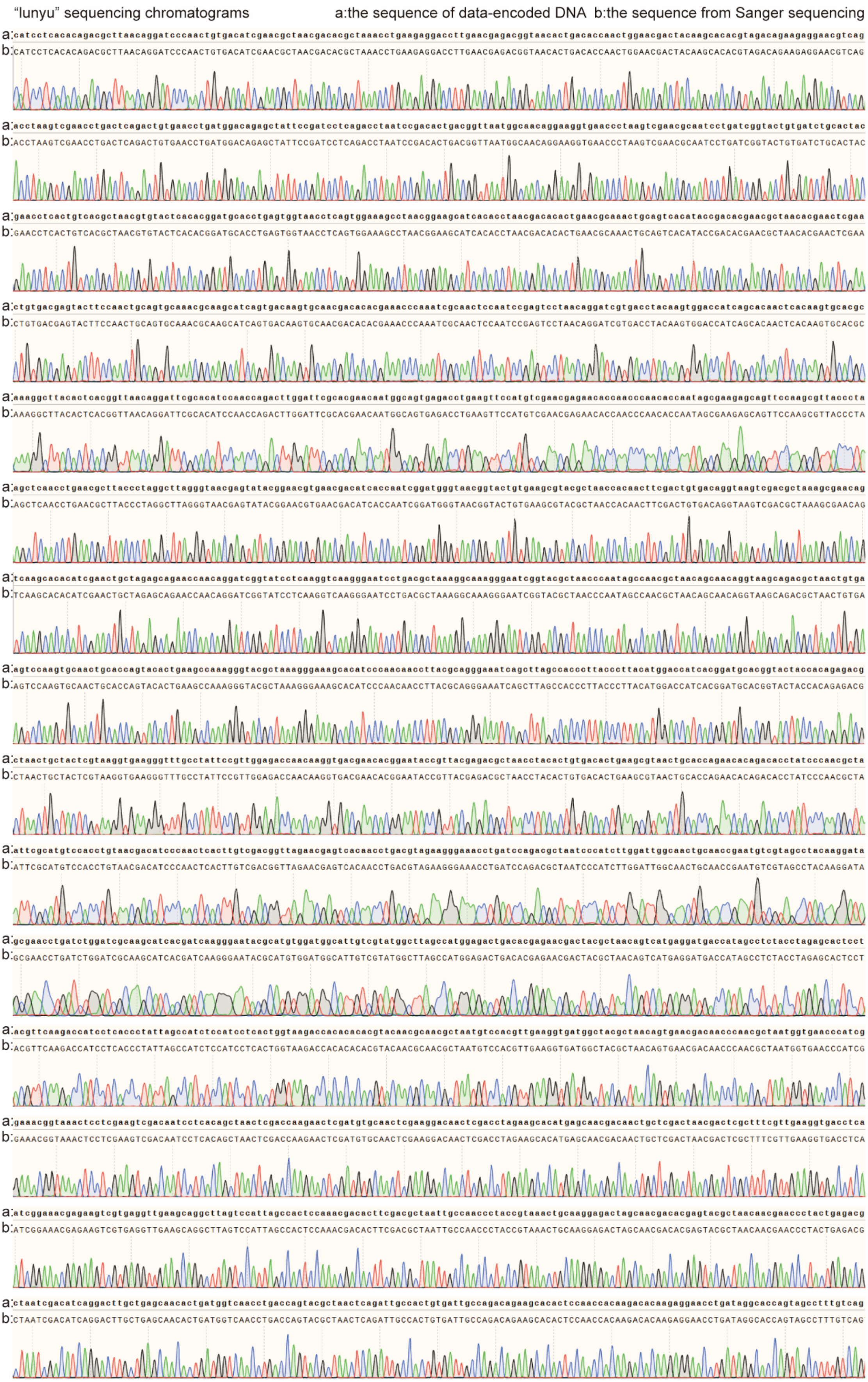
"lunyu" sequencing chromatograms
a:the sequence of data-encoded DNA b:the sequence from Sanger sequencing

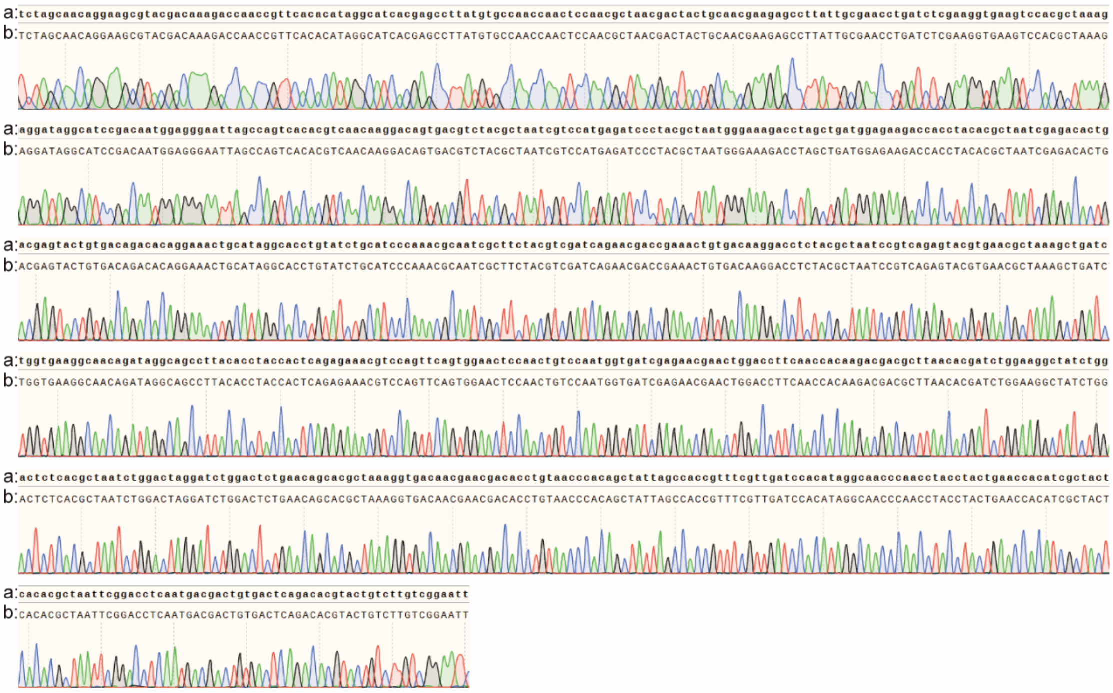

"cry" sequencing chromatograms a:the sequence of data-encoded DNA b:he sequence from Sanger sequencing

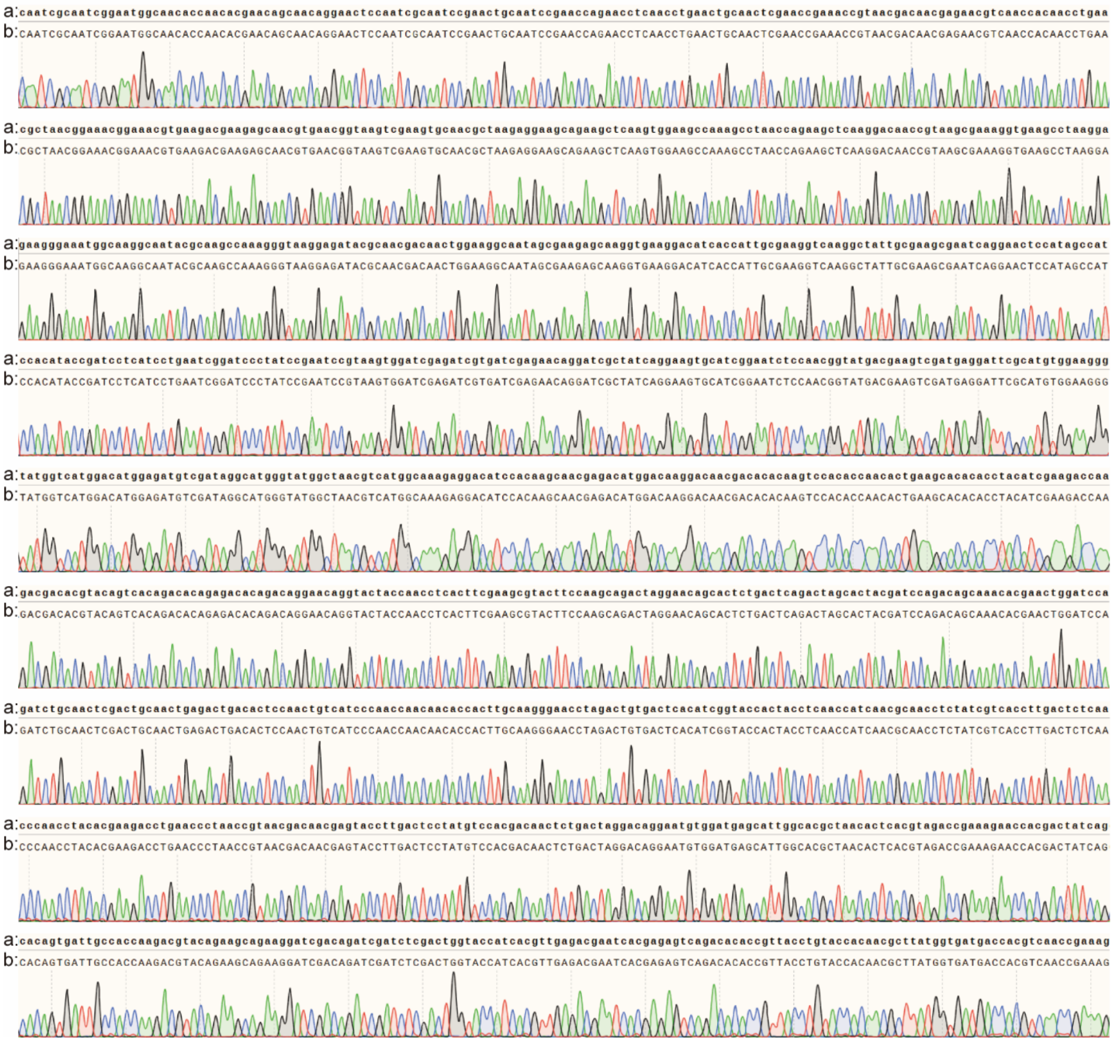

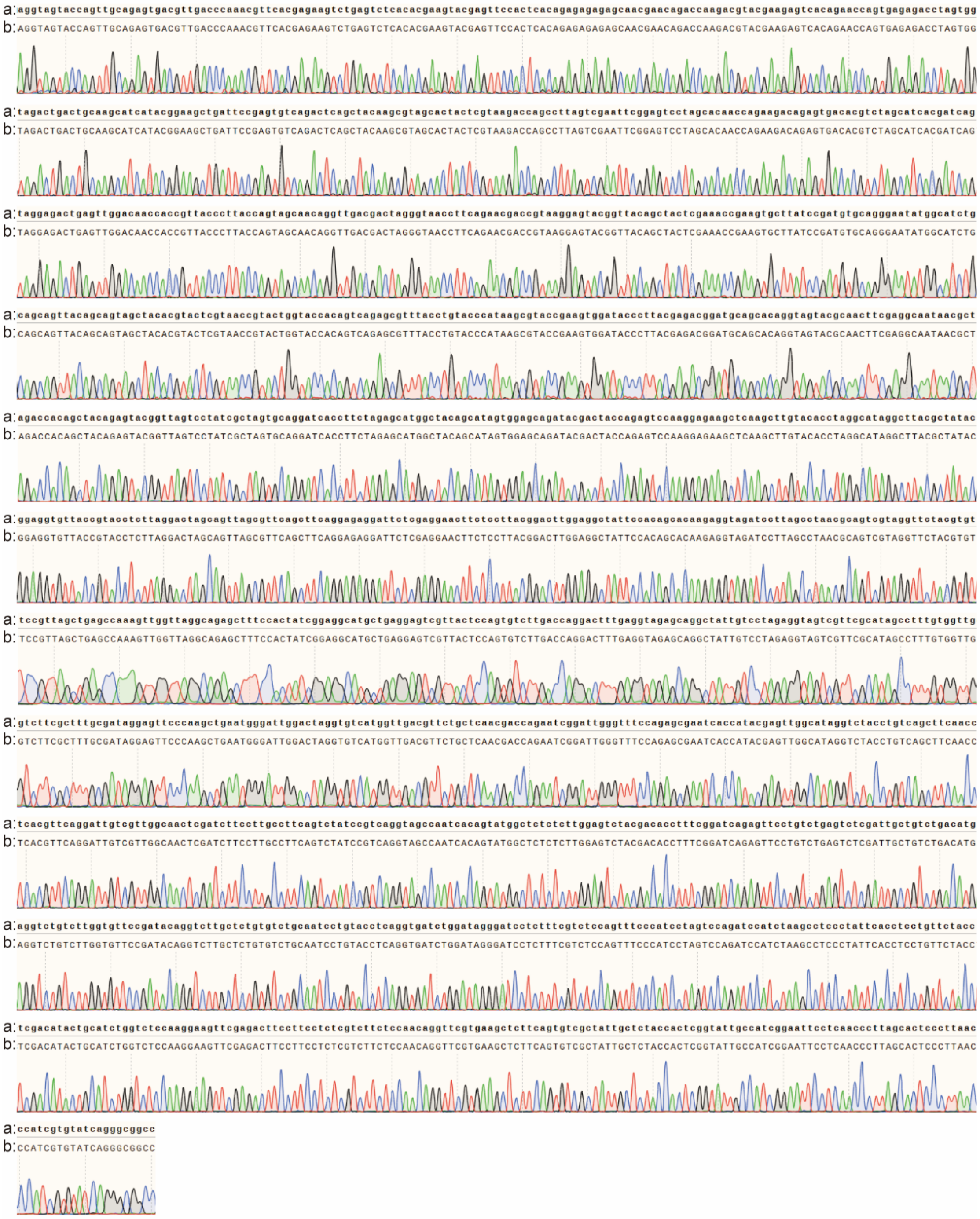

“lzw” sequencing chromatograms a:the sequence of data-encoded DNA b:the sequence from Sanger sequencing

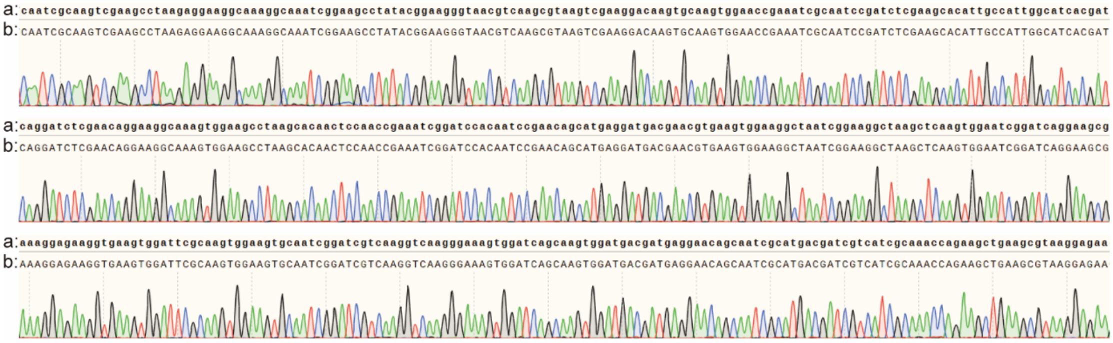

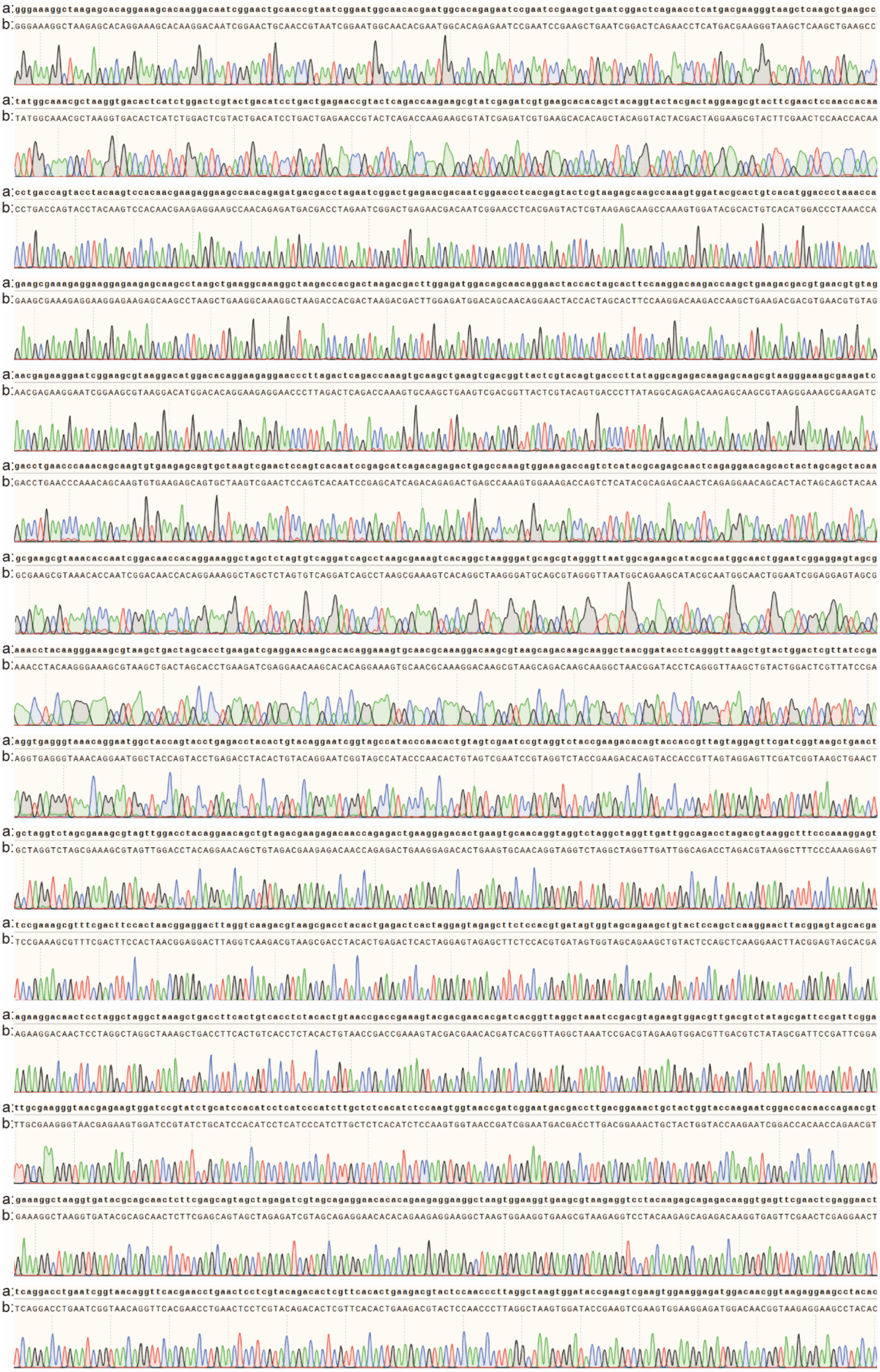
a:gggaaaggctaagagcacaggaaagcacaaggacaatcggaactgcaaccgtaatcggaatggcaacacgaatggcacagagaatccgaatccgaagctgaatcggactcagaacctcatgacgaagggtaagctcaagctgaagcc
b:GGGAAAGGCTAAGAGCACAGGAAAGCACAAGGACAATCGGAACTGCAACCGTAATCGGAATGGCAACACGAATGGCACAGAGAATCCGAATCCGAAGCTGAATCGGACTCAGAACCTCATGACGAAGGGTAAGCTCAAGCTGAAGCC
a:tatggcaaacgctaaggtgacactcatctggactcgtactgacatcctgactgagaaccgtactcagaccaagaagcgtatcgagatcgtgaagcacacagctacaggtactacgactaggaagcgtacttcgaactccaaccacaa
b:TATGGCAAACGCTAAGGTGACACTCATCTGGACTCGTACTGACATCCTGACTGAGAACCGTACTCAGACCAAGAAGCGTATCGAGATCGTGAAGCACACAGCTACAGGTACTACGACTAGGAAGCGTACTTCGAACTCCAACCACAA
a:cctgaccagtacctacaagtccacaacgaagaggaagccaacagagatgacgacctagaatcggactgagaacgacaatcggaacctcacgagtactcgtaagagcaagccaaagtggatacgcactgtcacatggaccctaaacca
b:CCTGACCAGTACCTACAAGTCCACAACGAAGAGGAAGCCAACAGAGATGACGACCTAGAATCGGACTGAGAACGACAATCGGAACCTCACGAGTACTCGTAAGAGCAAGCCAAAGTGGATACGCACTGTCACATGGACCCTAAACCA
a:gaagcgaaagaggaaggagaagagcaagcctaagctgaaggcaaaggctaagaccacgactaagacgacttggagatggacagcaacaggaactaccactagcacttccaaggacaagaccaagctgaagacgacgtgaacgtgtag
b:GAAGCGAAAGAGGAAGGAGAAGAGCAAGCCTAAGCTGAAGGCAAAGGCTAAGACCACGACTAAGACGACTTGGAGATGGACAGCAACAGGAACTACCACTAGCACTTCCAAGGACAAGACCAAGCTGAAGACGACGTGAACGTGTAG
a:aacgagaaggaatcggaagcgtaaggacatggacacaggaagaggaacccttagactcagaccaaagtgcaagctgaagtcgacggttactcgtacagtgacccttataggcagagacaagagcaagcgtaagggaaagcgaagatc
b:AACGAGAAGGAATCGGAAGCGTAAGGACATGGACACAGGAAGAGGAACCCTTAGACTCAGACCAAAGTGCAAGCTGAAGTCGACGGTTACTCGTACAGTGACCCTTATAGGCAGAGACAAGAGCAAGCGTAAGGGAAAGCGAAGATC
a:gacctgaacccaaacagcaagtgtgaagagcagtgctaagtcgaactccagtcacaatccgagcatcagacagagactgagccaaagtggaaagaccagtctcatacgcagagcaactcagaggaacagcactactagcagctacaa
b:GACCTGAACCCAAACAGCAAGTGTGAAGAGCAGTGCTAAGTCGAACTCCAGTCACAATCCGAGCATCAGACAGAGACTGAGCCAAAGTGGAAAGACCAGTCTCATACGCAGAGCAACTCAGAGGAACAGCACTACTAGCAGCTACAA
a:gcgaagcgtaaacaccaatcggacaaccacaggaaaggctagctctagtgtcaggatcagcctaagcgaaagtcacaggctaagggatgcagcgtagggttaatggcagaagcatacgcaatggcaactggaatcggaggagtagcg
b:GCGAAGCGTAAACACCAATCGGACAACCACAGGAAAGGCTAGCTCTAGTGTCAGGATCAGCCTAAGCGAAAGTCACAGGCTAAGGGATGCAGCGTAGGGTTAATGGCAGAAGCATACGCAATGGCAACTGGAATCGGAGGAGTAGCG
a:aaacctacaagggaaagcgtaagctgactagcaccttgaagatcgaggaacaagcacacaggaaagtgcaacgcaaaggacaagcgtaagcagacaagcaaggctaacggatacctcagggttaagctgtactggactcgttatccga
b:AAACCTACAAGGGAAAGCGTAAGCTGACTAGCACCTTGAAGATCGAGGAACAAGCACACAGGAAAGTGCAACGCAAAGGACAAGCGTAAGCAGACAAGCAAGGCTAACGGATACCTCAGGGTTAAGCTGTACTGGACTCGTTATCCGA
a:aggtgagggtaaacaggaatggctaccagtacctgagacctacactgtacaggaatcggtagccatacccaacactgtagtcgaatccgtaggtctaccgaagacacagtaccaccgttagtaggagttcgatcggtaagctgaact
b:AGGTGAGGGTAAACAGGAATGGCTACCAGTACCTGAGACCTACACTGTACAGGAATCGGTAGCCATACCCAACACTGTAGTCGAATCCGTAGGTCTACCGAAGACACAGTACCACCGTTAGTAGGAGTTCGATCGGTAAGCTGAACT
a:gctaggtctagcgaaagcgtagttggacctacaggaacagctgtagacgaagagacaaccagagactgaaggagacactgaagtgcaacaggtaggtctaggctaggttgattggcagacctagacgtaaggctttcccaaaggagt
b:GCTAGGTCTAGCGAAAGCGTAGTTGGACCTACAGGAACAGCTGTAGACGAAGAGACAACCAGAGACTGAAGGAGACACTGAAGTGCAACAGGTAGGTCTAGGCTAGGTTGATTGGCAGACCTAGACGTAAGGCTTTCCCAAAGGAGT
a:tccgaaagcgtttcgacttccactaacggaggacttaggtcaagacgtaagcgacctacactgagactcactaggagtagagcttctccacgtgatagtggtagcagaagctgtactccagctcaaggaacttacggagtagcacga
b:TCCGAAAGCGTTTCGACTTCCACTAACGGAGGACTTAGGTCAAGACGTAAGCGACCTACACTGAGACTCACTAGGAGTAGAGCTTCTCCACGTGATAGTGGTAGCAGAAGCTGTACTCCAGCTCAAGGAACTTACGGAGTAGCACGA
a:agaaggacaactcctaggctaggctaaagctgaccttcactgtcacctctacactgtaaccgaccgaaagtacgacgaacacgatcacggttaggctaaatccgacgtagaagtggacgttgacgtctatagcgattccgattcgga
b:AGAAGGACAACTCCTAGGCTAGGCTAAAGCTGACCTTCACTGTCACCTCTACACTGTAACCGACCGAAAGTACGACGAACACGATCACGGTTAGGCTAAATCCGACGTAGAAGTGGACGTTGACGTCTATAGCGATTCCGATTCGGA
a:ttgcgaagggtaacgagaagtggatccgtatctgcatccacatcctcatcccatcttgctctcacatctccaagtggtaaccgatcggaatgacgaccttgacggaaactgctactggtaccaagaatcggaccacaaccagaacgt
b:TTGCGAAGGGTAACGAGAAGTGGATCCGTATCTGCATCCACATCCTCATCCCATCTTGCTCTCACATCTCCAAGTGGTAACCGATCGGAATGACGACCTTGACGGAAACTGCTACTGGTACCAAGAATCGGACCACAACCAGAACGT
a:gaaaggctaaggtgatacgcagcaactcttcgagcagtagctagagatcgtagcagaggaacacacagaagaggaaggctaagtggaaggtgaagcgtaagaggtcctacaagagcagagacaaggtgagttcgaactcgaggaact
b:GAAAGGCTAAGGTGATACGCAGCAACTCTTCGAGCAGTAGCTAGAGATCGTAGCAGAGGAACACACAGAAGAGGAAGGCTAAGTGGAAGGTGAAGCGTAAGAGGTCCTACAAGAGCAGAGACAAGGTGAGTTCGAACTCGAGGAACT
a:tcaggacctgaatcggtaacaggttcacgaacctgaactcctcgtacagacactcgttcacactgaagacgtactccaaccccttaggctaagtggataccgaagtcgaagtggaaggagatggacaacggtaagaggaagcctacac
b:TCAGGACCTGAATCGGTAACAGGTTCACGAACCTGAACTCCTCGTACAGACACTCGTTCACACTGAAGACGTACTCCAACCCCTTAGGCTAAGTGGATACCGAAGTCGAAGTGGAAGGAGATGGACAACGGTAAGAGGAAGCCTACAC

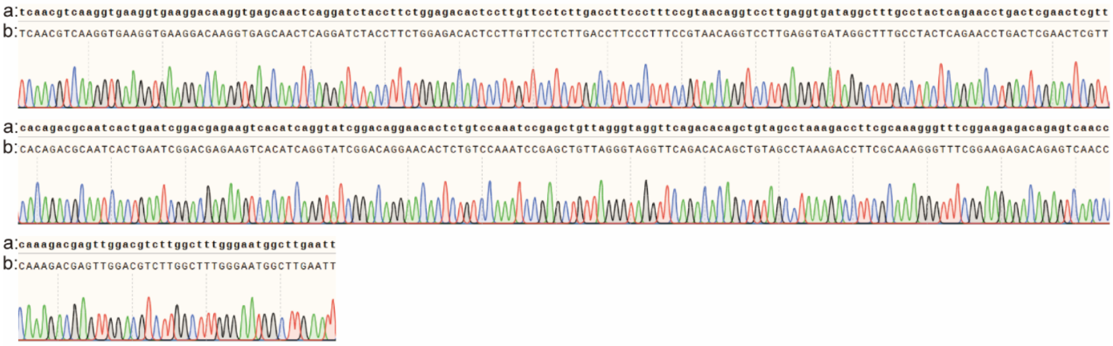

"panda" sequencing chromatograms a:the sequence of data-encoded DNA b:the sequence from Sanger sequencing

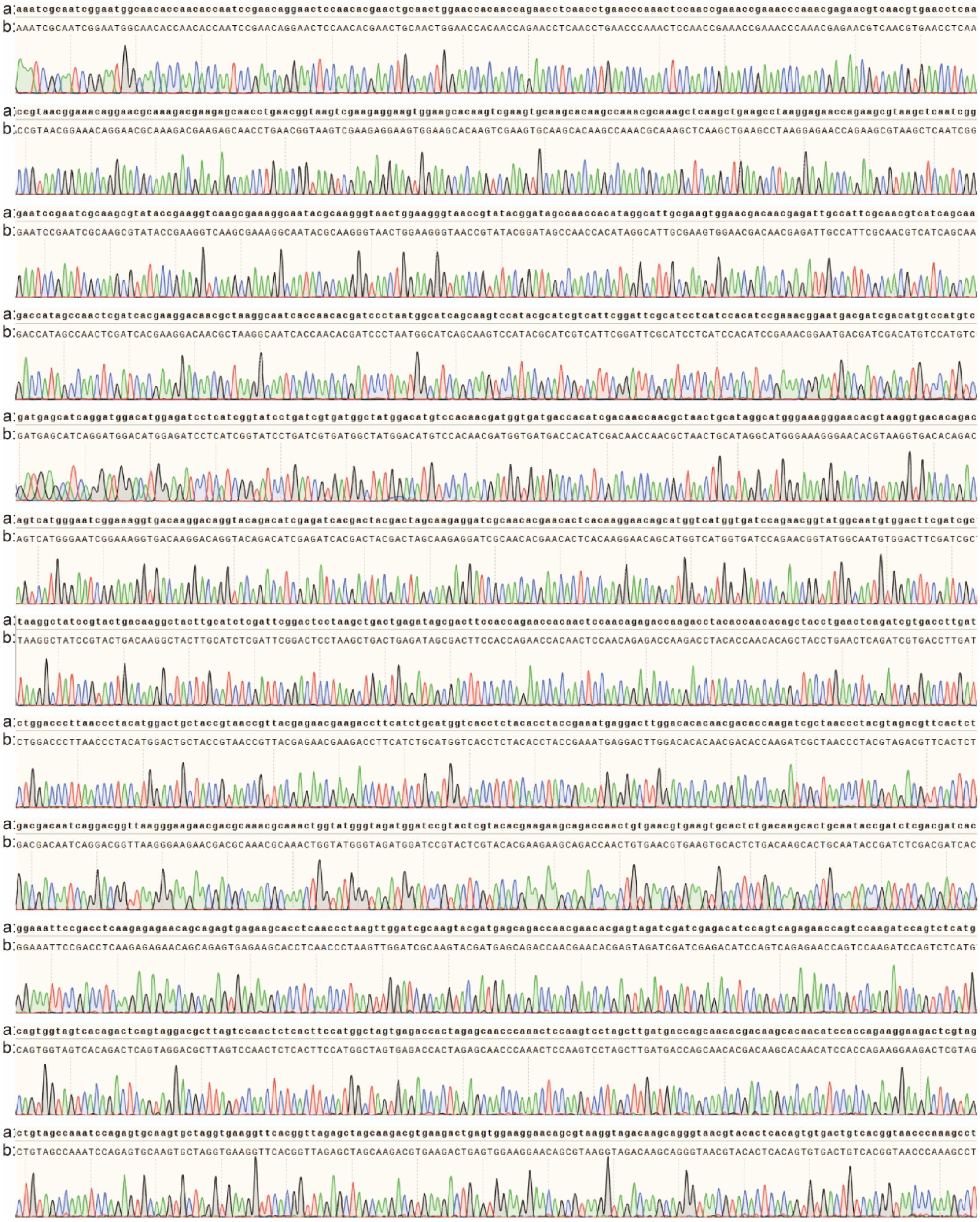

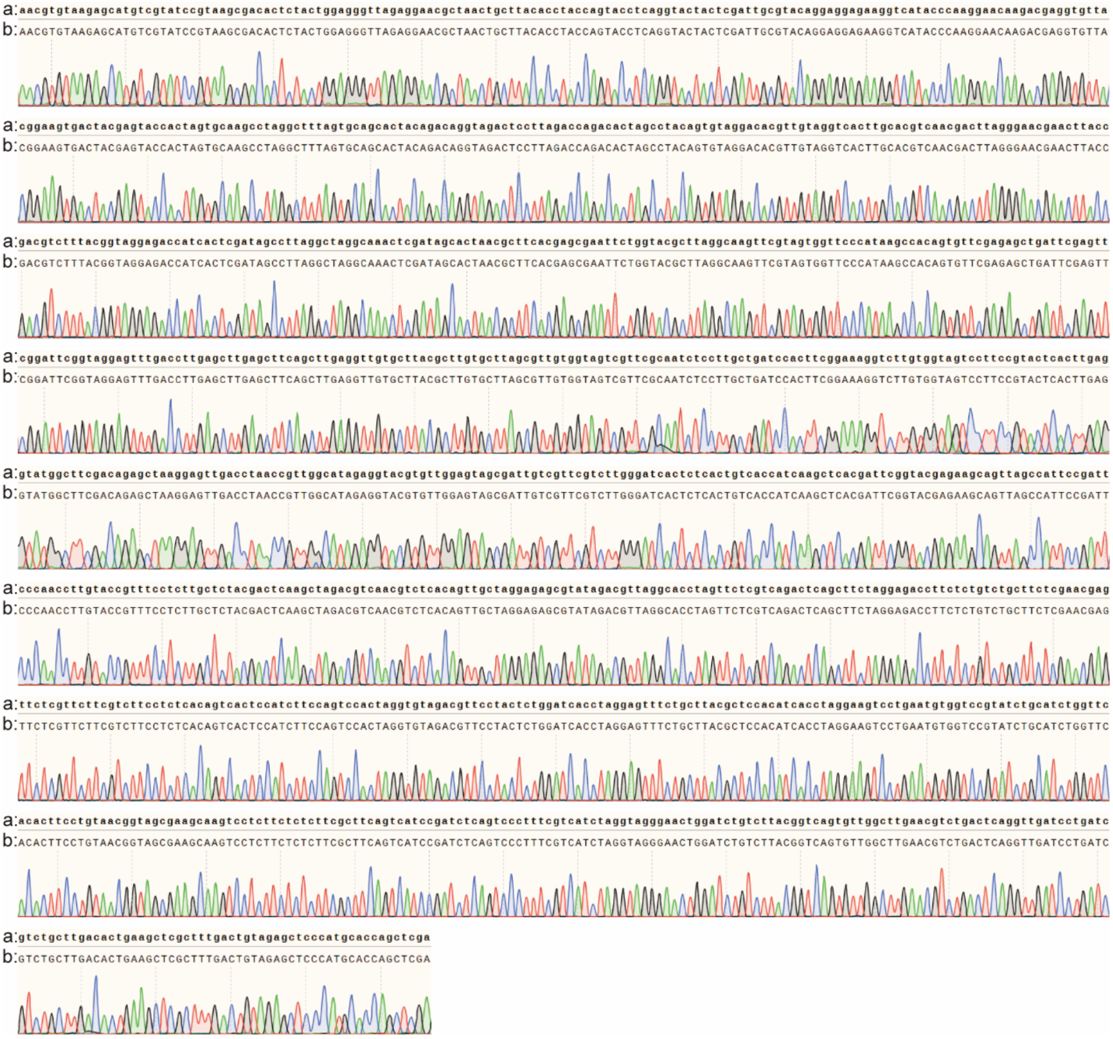

Figure S7. DNA sequencing validation of information-encoded DNA retrieved from the cell pool of the 100th generation.

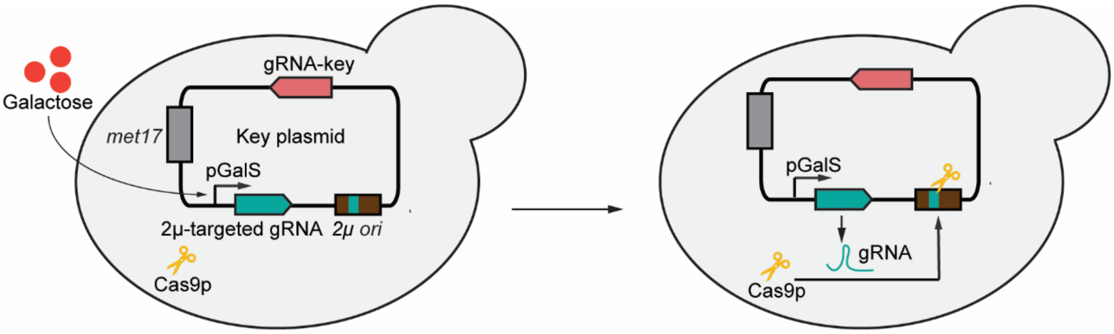

Figure S8. Schematic diagram of galactose-induced elimination of the gRNA "key" plasmid. In the gRNA key plasmid, a galactose-inducible gRNA expression cassette was preinstalled and would express a gRNA that guides Cas9p to cleave and eradicate the plasmid efficiently upon galactose induction.

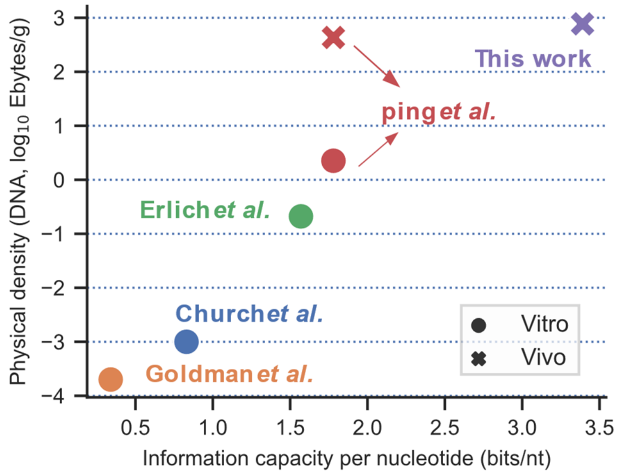

Figure S9. Physical density comparison among different DNA data storage algorithms. The algorithm by Ping *et al.*,[4] which exhibited the previous maximum level of physical density, was included in the comparison. Algorithms proposed by Church *et al.*,[1] Goldman *et al.*,[2] and Erlich *et al.*.[3] were also included for the analysis. With the loss-compression ability, "Panda" stores the five digital file in this study with an average information density (information capacity per nucleotide) of 3.39 bits/nt. On top of this information density, Panda could achieve the highest physical density. The calculating process is referred to Ping *et al.*.[4]

Supplementary tables

Table S1. Comparison between "Panda" and other well-known DNA data storage algorithms

Refs	Binary conversion[1]	Compression function[2]	Bio-constraints Control[3]	Redundancy[4]	Error correction[5]	Tested data (Mbytes)[6]	Full Recovery[7]	Information Density (Information capacity per nucleotide; bits/nt)[8]
Church *et al.*[1]	Needed	No	Homopolymer	1	No	0.65	NO	0.83
Goldman *et al.*[2]	Needed	No	Homopolymer	4	Yes	0.75	NO	0.34
Grass *et al.*[5]	Needed	No	No	1	Yes	0.08	Yes	1.19
Bornholt *et al.*[6]	Needed	No	Homopolymer	1.5	No	0.15	No	0.88
Blawat *et al.*[7]	Needed	No	Homopolymer	1.13	Yes	22	Yes	0.92
Erlich *et al.*[3]	Needed	No	GC content, homopolymer	1.07	Yes	2.15	Yes	1.57
Yazdi *et al.*[8]	Needed	No	GC content, homopolymer	1.18	Yes	$9.72*10^{-3}$	Yes	1.74
Organick *et al.*[9]	Needed	No	GC content, homopolymer	1.15	Yes	over 200	Yes	1.10
Melpomeni Dimopoulou *et al.*[10]	Unnecessary	Yes	GC content, homopolymer	1	No	0.26	Yes	1.71
Press *et al.*[11]	Needed	No	GC content, homopolymer	1.67	Yes	2	Yes	1.20
Chen *et al.*[12]	Needed	No	No	1.2	Yes	0.037	Yes	1.19
Ping *et al.*[4]	Needed	No	GC content, homopolymer	1.25	Yes	0.25	No	1.75-1.78
This work	Unnecessary	Yes	GC content, homopolymer	1	No	$6.29*10^{-3}$	Yes	3.39 (Compression ratio: 2.19[#])

[1]"Binary conversion" refers to whether the encoding process involves a binary conversion step.

[2]"Compressed function" refers to whether the algorithm integrates a compressed function.

[3]"Bio-constraint control" refers to whether the algorithm controls the GC content and homopolymer length of DNA sequences.

[4]"Redundancy" refers to the number of times the output information is compared to the input information.

[5]"Error correction" refers to whether the algorithm adds error correction codes.

[6] "Tested data" refers to the practical data storage into synthetic DNA by the algorithms.

[7] "Full recovery" refers to whether the data stored in the synthetic DNA is recovered completely.

[8] "Information Density (Information capacity per nucleotide)" refers to the average quantity of information in one nucleotide that calculated from practical data storage in the synthetic DNA.

[#] The average compression ratio of five practical files we stored by "Panda" in this study is presented.

Table S2. Primers and Plasmids used in this work

Systematic Name	Name	Sequence	Description
ZHO306	sequencing-F	TACCAGTACGTTATGCCTAT	Sequencing primers
ZHO307	sequencing-R	AGCAACACGTACTTCGAAAT	
ZHO308	panda	GACTGTCTGTGTCACCTTAC	Specific primers on DNA information
ZHO309	smile	GTCTGTGACTGTGGTCTTAG	
ZHO314	lunyu	AGCTTAGGGTAACGCTTGG	
ZHO315	cry	CGTCTTGGTCTTCGATGTAG	
ZHO317	lzw	AGTACGCTTCCTAGTCGTAG	
PZH187	lock1_*ura3*	-	Lock_*ura3* plasmid
PZH189	lock2_*ura3*	-	
PZH193	lock3_*ura3*	-	
PZH197	lock4_*ura3*	-	
PZH221	lock1_*leu2*	-	Lock_*leu2* plasmid
PZH232	lock2_*leu2*	-	
PZH233	lock3_*leu2*	-	
PZH234	lock4_*leu2*	-	
PZH223	key1	-	Key plasmid
PZH227	key2	-	
PZH228	key3	-	
PZH229	key4	-	
PZH238	PRS413_lunyu	-	DNA information plasmid*
PZH239	PRS413_lzw	-	
PZH240	PRS413_panda	-	
PZH241	PRS413_smile	-	
PZH242	PRS413_cry	-	

*The sequences of DNA information are listed in Note S2.

Table S3. Strains used in this work

Systematic Name	Genotype
BY4741	*MATa his3Δ1 leu2Δ0 met15Δ0 ura3Δ0*
ZHY277	BY4741 *ho*::pGAP-cas9-tCYC1
ZHY136	ZHY277 ChrIX::lock1_*ura3* PRS413-lzw
ZHY137	ZHY277 ChrIX::lock2_*ura3 ade2*Δ0 PRS413-panda
ZHY186	ZHY277 ChrIX::lock1_*ura3* ChrIII::lock1_*leu2* PRS413-lzw
ZHY187	ZHY277 ChrIX::lock2_*ura3* ChrIII::lock2_*leu2 ade2*Δ0 PRS413-panda
ZHY211	ZHY277 ChrIX::lock2_*ura3* ChrIII::lock2_*leu2* PRS413-panda
ZHY212	ZHY277 ChrIX::lock3_*ura3* ChrIII::lock3_*leu2* PRS413-smile
ZHY213	ZHY277 ChrIX::lock4_*ura3* ChrIII::lock4_*leu2* PRS413-lunyu
ZHY214	ZHY277 ChrIX::lock3_*ura3* ChrIII::lock3_*leu2* PRS413-cry

Table S4. Different type files encoded by "Panda"

File type[1]	File name	File size (KB)	Compression ratio[2]	Transcoding efficiency (bits/nt)[3]	Information Density (Information capacity per nucleotide) (bits/nt)
	lunyu.txt	1.78	2.92 #	1.67	4.87
	A_Dream_in_Red_Mansions.txt	2513.82	2.80	1.58	4.43
	Notre_Dame_de_Paris.txt	1201.61	2.61	1.66	4.35
	The_Count_of_Monte_Cristo.txt	2721.85	2.73	1.58	4.31
	Journey_to_the_west.txt	2108.83	2.67	1.58	4.22
	Anna_Karenina.txt	2019.51	2.64	1.58	4.18
	Origin_of_Species-Charles_Darwin.txt	541.61	2.62	1.57	4.12
	Don_Quijote_de_la_Mancha.txt	1060.39	2.45	1.66	4.07
**Text**	Wuthering_Heights.txt	677.62	2.31	1.66	3.84
	Madame_Bovary.txt	678.70	2.30	1.66	3.83
	Hamlet_Prince_of_Denmark.txt	202.18	2.17	1.66	3.61
	PMC8928933_PMC8860355.txt_10000_5.txt	9.79	1.91	1.66	3.17
	PMC8928933_PMC8860355.txt_9000_6.txt	8.81	1.91	1.66	3.17
	PMC8928933_PMC8860355.txt_10000_6.txt	9.78	1.86	1.66	3.09
	PMC8928933_PMC8860355.txt_8000_7.txt	7.82	1.83	1.67	3.05
	PMC8928933_PMC8860355.txt_10000_2.txt	9.77	1.80	1.66	3
	lzw.txt	1.05	1.74 #	1.67	2.89
	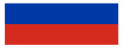	9760.72	129.40	1.66	215.43
**Image**	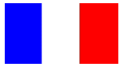	9767.27	69.95	1.57	109.97
	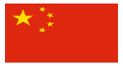	732.40	32.40	1.66	53.94

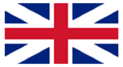	976.12	28.38	1.56	44.29
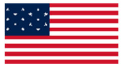	976.12	26.07	1.56	40.68
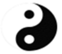	111.39	11.16	1.55	17.24
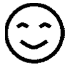	1.20	2.18 #	1.50	3.28
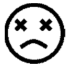	1.13	2.04 #	1.50	3.06
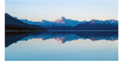	3798.05	1.82	1.66	3.03
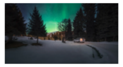	3798.05	1.77	1.66	2.94
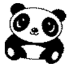	1.06	1.89 #	1.50	2.83
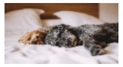	9760.72	1.50	1.66	2.49
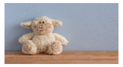	9760.72	1.45	1.66	2.41
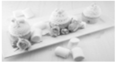	407.48	1.39	1.66	2.31
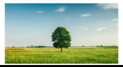	3709.04	1.35	1.59	2.14

[1]Two different file types (text files and images) were used to test the practical data encoding by “Panda” algorithm. The information density (information capacity per nucleotide) of much data is more than 3 bits/nt.

[2]The compression ratio is obtained by dividing the number of bits in the original data before compression by the number of bits in the data after compression following the pipeline depicted in Figure S3.

[3]The transcoding efficiency is calculated by dividing the number of bits in the data after compression by the number of nucleotides after encoding following the pipeline depicted in Figure S3.

[#]The compression ratio of each practical file we stored by “Panda” in this study is presented.

Supporting Information Text

Note S1:

The detailed computing procedure of Panda algorithm

In order to store practical data into the "cell hard disk", we devised an codec algorithm, named "Panda". It encodes data without binary conversion and merges data compression with the data codec process, simplifying the data codec steps and allowing DNA data storage to be not limited to present binary systems. It achieves lossless "LZW" data compression on top of DNA-based data storage while controlling GC and homopolymer length to an acceptable level at the encoded DNA sequence, making them be suitable for downstream biochemical working flow. Specifically, it encodes DNA with a regional GC content of roughly 50% and a homopolymer run length of less than 4 nt, which facilitates long gene synthesis, commonly utilized for *in vivo* data storage, such as our "cell hard drive". The details of the algorithm are presented below.

To begin, the elements consisted of the specific data were extracted. For text, characters and symbols composition were taken as elements. For image, the RGB value of each pixel (0-255) were firstly extracted and ordered as a "number" text with number "0-9" taken as "elements". These elements form a "set", as represented by "S_0" Eq. (1). The total number of elements are also recorded as represented by "i" Eq. (1). Further, element composition of S_0 is counted and forms a set, S_1 Eq. (2), where "a" represents non-repeated element, "n" represents the number of times the corresponding element has been repeated, and "m" represents the index of different elements.

$$S_0 = \{e_1, e_2, \ldots, e_i\} \quad (i \in \mathbb{N}^+) \tag{1}$$

$$S_1 = \{n_1 \times a_1, n_2 \times a_2, \ldots, n_m \times a_m\} \quad (m, n \in \mathbb{N}^+) \tag{2}$$

Further, the information sequence is deduplicated, resulting in the following set, S_{Set}:

$$S_{Set} = \{a_1, a_2, \ldots, a_m\} \tag{3}$$

We further define the code table based on S_{Set}. A sequence consisting of all mapped values in the code table following an Arithmetic progression, as shown below:

$$b_n = b_1 + (n-1)\alpha \tag{4}$$

Where b_n represents the mapping value of the nth position in the final code table "M", "α" represents the tolerance of the Arithmetic progression, and "b_1" represents the first item of the Arithmetic progression. The mapping values of the complete code table is then obtained while defining $\alpha = 1$, $b_1 = 1$. Furthermore, the code table is initialized based on Eq. (3) and Eq. (4):

$$M = \{a_1 \to 1, a_2 \to 2, \cdots, a_m \to b_m\} \tag{5}$$

Subsequently, we encode S_0 and synchronously expand the code table. The following is a general expression for any cycle in the encoding process.

We started from the position j in the S_0 and search for the longest key that can match in the code table for mapping. The mapped value is recorded as d, which is a decimal value. If the key length is marked as l, the new key to be added is K :

$$K = \{e_j, e_{j+1,\ldots,} e_{j+l}\} \tag{6}$$

and its length is "$l+1$"。

The starting point for the new round of encoding is e_{j+l}, and the above steps are repeated until the sequence is completely encoded. Finally, we code the sequence as a sequence composed of Decimal values, which is recorded as D:

$$D = \{d_0, d_1, \ldots, d_\xi\} \tag{7}$$

Next，we performed a decimal conversion, and the result is recorded as q ,

$$q = \{n_i, n_{i-1}, \ldots, n_1\} \tag{8}$$

where "n" represents the number and "i" represents the order. Moreover, n_i meets the following:

$$n_i = \frac{d}{\mu^{i-1}} \% \mu \quad (d \geq \mu^{i-1}) \tag{9}$$

where μ (μ=4) represents the base number converted from decimal number.

So far, we have compressed and converted the element sequence S_0 of the original information to obtain the Quaternary number sequence S_q

$$S_q = \{q_1, q_2, \dots, q_\xi\} \quad (10)$$

Then, we convert the Quaternary number to the base sequence. There are 24 kinds of this mapping relationship (4!). In this paper, we choose one of them to map the Quaternary number sequence to the base sequence:

$$M_{base} = \{0 \to A, 1 \to T, 2 \to C, 3 \to G\} \quad (11)$$

Furthermore, in order to make the encoded sequence match the following biochemical working flow, we employed a two-step extension process as described in our previous study. Briefly, we divided the obtained base sequence into units with a length of 5 nt, and extended each unit to a unit with a length of 6 nt, that is controlled with a GC ratio approaching 50%. Next, "ATGCA", "ATGCT", "ATGCG" and "ATGCC" are used to replace "AAAA", "TTTT", "GGGG" and "CCCC" in the extended sequence from the first step, eliminating homopolymer sequence with the length equal or above 4 nt.

To this end, we have completed the compression encoding of the original information sequence, and controlled the GC content and homopolymer runs in the sequence.

About "panda" algorithm

Current DNA data storage algorithms, such as those proposed by Church *et al.*,[1] Goldman *et al.*,[2] Erlich *et al.*[3] and Ping *et al.*[4] all use a binary conversion procedure. This could limit "DNA data storage" to the binary computational realm, which does not appear to fully utilize DNA's "quaternary" characteristic. Given that "DNA" is a pure "quaternary" medium and that the DNA-based storage "disk" may be employed in future DNA computation and more diverse applications (a camera, for example, can capture a picture and store it straight on the "quaternary" DNA-based "disk."), it is of broad importance to create an algorithm that skips the binary conversion stage. We earlier developed such an algorithm based on "quaternary" Huffman coding.[13] However, "Huffman" logic necessitates the storage of a large code table, that is tedious and might be losed over a lengthy period of storage. As a result, we developed the

“panda” algorithm using “LZW” logic. The lossless compression method “LZW” is widely employed in silicon-based computer data storage. It does not require the large coding table to be stored in the output. The “panda” algorithm uses “LZW” logic to reduce the encoded DNA bases, minimizing the cost of DNA synthesis for data storage. Specifically, this algorithm encodes DNA with a regional GC content of roughly 50% and a homopolymer run length of less than 4 nt. This enables “panda” be better than many other well-validated DNA data storage algorithms, e.g. Church *et al.*,[1] Goldman *et al.*,[2] Erlich *et al.*[3] and Ping *et al.*[4] where bio-constraint control is only performed at the whole sequence level, in storing information using long gene, given that long gene synthesis generally requires a strict control of sequence homogeneity at regional level since assembly of multiple oligonucleotides into one gene by PCR-based method might be affected by regional GC level. Overall, the “panda” algorithm described here encodes data without binary conversion and merges data compression with the data codec process. This should simplify the data codec steps and allow DNA data storage to be not limited to present binary systems. It achieves lossless “Lempel-Ziv Welch (LZW)” data compression[14] on top of DNA-based data storage while controlling GC and homopolymer length to an acceptable level at the encoded DNA sequence, making them be suitable for downstream biochemical working flow. Owing to the high density and good biochemical control of “panda”, it should facilitate the information storage into the “Cell Disk”.

Calculation of information density of “Panda” algorithm

Since “Panda” is a lossless compression algorithm, its information density changes as data changes. As an example, we calculated the average density of the practical files we stored by “Panda” in this study. The original data was first compressed in the algorithm, as depicted in Figure S3, with the compression ratio (R) represented as follows:

$$R = \frac{I}{I_c} \tag{12}$$

where I denotes the size of the original data and I_c denotes the size of the data after "Panda" compression.

The compressed data was then subjected to transcoding processes, as depicted in Figure S3, with transcoding efficiency (E) represented as follows:

$$E = \frac{I_c}{N} \tag{13}$$

where N denotes the number of nucleotides of DNA sequences.

The information density can therefore be determined as follows:

$$d = E \times R = \frac{I}{N} \tag{14}$$

The average information density can then be expressed as:

$$\bar{d} = \frac{I'}{N'} \tag{15}$$

whereas I' and N' are represented as follows:

$$I' = \sum_{a=1}^{j} I_a \tag{16}$$

$$N' = \sum_{b=1}^{j} N_b \tag{17}$$

where j is the number of samples used to calculate the average information density in Eqs. (16) and (17).

As shown in Table S1, we utilize "Panda" to encrypt a total of 5 files, of which, "smile.bw.bmp", "upset.bw.bmp", and "panda.bw.bmp" are black and white image files, with sizes of 9616 bits, 9040 bits, and 8464 bits; and "lzw.txt" and "lunyu.txt" are text files with sizes of 8616 bits and 14576 bits, respectively. In terms of Eqs. (15-17), the average information density of these five files is calculated as following:

$$\bar{d} = \frac{8616 + 14576 + 9616 + 9040 + 8464}{2977 + 2996 + 1934 + 2954 + 2989} \approx 3.39\text{bits/nt} \tag{18}$$

Projection of density and cost of "Cell Disk"

As a new storage system, the density and cost of "Cell Disk" is projected here. Given that one yeast cell can retain up to 1 Mb of exogeneous DNA, we proposed a "Cell Disk 1" prototype with 10^5 cells, each holding 1 Mb of data stored DNA. Similarly,

because it has been demonstrated that a 254 kB artificial chromosome containing stored texts and images can be duplicated in the yeast cell,[12] we proposed a "Cell Disk 2" prototype including 10^5 cells, each of which contains 254 kb data stored DNA. The total data ($I_{carrier}$) recorded on the disk is then determined using the formula below:

$$I_{carrier} = d \times L_{DNA} \times n_{cell} \tag{19}$$

where d represents "information density", L_{DNA} represents the length of the DNA, n_{cell} represents the number of cells.

As a result, the total data（$I_{carrier}$) stored on "Cell Disk 1" is calculated to be "39.465 GB", whereas the total data stored on "Cell Disk 2" is calculated to be "10.255 GB".

The density of "Cell Disk" is determined by the quantity of information carried by the carrier per unit mass, like with other disks such as soft disk, light disk, flash memory, and hard disk.

$$D = \frac{I_{carrier}}{m_{carrier}} \tag{20}$$

where $I_{carrier}$ stands for information on the quantity of carrier carried, and $m_{carrier}$ stands for the mass of carrier.

As illustrated in Fig. 5, the mass of each yeast cell is estimated to be 98 pg, which is the medium value of the range of yeast cell mass.[15] Therefore, the density of "Cell Disk 1" is computed to be 4.324×10^{15} bytes/g and the density of "Cell Disk 2" is computed to be 1.046×10^{15} bytes/g. Other disks' masses are acquired from commercially available websites, including a hard disk weighing 200 g (type: WDBYVGO010BWT), flash memory weighing 30 g (type: aigo-U330), soft disk weighing 36 g (type: SONY-10MFD2HDCFM 2HD), and light disk weighing 20 g (type: Verbatim- VBR520YP20SD4). Referred to their capacity, the densities of these disks are computed to be 5.498×10^9 bytes/g (hard disk), 9.163×10^9 bytes/g (flash memory), 41943 bytes/g (soft disk) and 5.369×10^9 bytes/g (light disk), respectively.

Following Moore's law,[16] the cost of retaining an equivalent quantity of information in an electronic component will drop by 50% every 18 months. While more

and more research efforts have been placed on DNA data storage, we anticipated the cost decrease of DNA synthesis will resemble other electronic components following Moore's law, in terms of below formula:

$$P_{base} = 4^{-\frac{t}{3}} \times \mathrm{P}_0 \tag{21}$$

where P_0 represents the current cost of one base and P_{base} represents the cost after t years. Given that the current DNA synthesis cost is quite high and it accounts for the major cost of DNA data storage, we used the cost change of total DNA synthesis by “Cell Disk” to simulate its cost development. Therefore, the cost of “Cell Disk” is calculated as following：

$$\mathrm{P}_{cell\ disk} = P_{base} \times n_{cell} \times L_{DNA} \tag{22}$$

where $\mathrm{P}_{cell\ disk}$ represents the price of Cell Disk,n_{cell} represents the number of yeast cells in Cell Disk, and L_{DNA} represents the length of DNA in Cell Disk.

According to Eq. 19, L_{DNA} can be denoted as:

$$L_{DNA} = \frac{I_{carrier}}{d \times n_{cell}} \tag{23}$$

Therefore, by the combination of Eqs. 21–23, the cost of Cell Disk at time can be calculated as following:

$$\mathrm{P}_{cell\ disk} = \frac{4^{-\frac{t}{3}} \times \mathrm{P}_0 \times I_{carrier}}{d} \tag{24}$$

Notably, as shown in Fig. 5, the cost of the synthetic gene in this work is \$0.07/base and d is valued as 3.39 bits/nt. Moreover, as a control, the cost of a DNA data storage system with d value of “2 bits/nt” is calculated following the same formula as $\mathrm{P}_{cell\ disk}$ and included in the figure. The current available price of hard disk (\$4.259 $\times$ 10^{-8} k/MB) is also included as a reference. It is estimated that the cost of “Cell Disk” might approach the cost of hard disk within 50 years.

Note S2:

Nucleotide sequences of “lock” plasmids

Nucleotide sequences of “lock_*ura3*” module

TTCAATTCAATTCATCATTTTTTTTTTATTCTTTTTTTTGATTTCGGTTTCTTTG
AAATTTTTTTGATTCGGTAATCTCCGAACAGAAGGAAGAACGAAGGAAGG
AGCACAGACTTAGATTGGTATATATACGCATATGTAGTGTTGAAGAAACATG
AAATTGCCCAGTATTCTTAACCCAACTGCACAGAACAAAAACCTGCAGGA
AACGAAGATAAATCATGTCGAAAGCTACATATAAGGAACGTGCTGCTACTC
ATCCTAGTCCTGTTGCTGCCAAGCTATTTAATATCATGCACGAAAAGCAAAC
AAACTTGTGTGCTTCATTGGATGTTCGTACCACCAAGGAATTACTGGAGTTA
GTTGAAGCATTAGGTCCCAAAATTTGTTTACTAAAAACACATGTGGATATCT
TGACTGATTTTTCCATGGAGGGCACAGTTAAGCCGCTAAAGGCATTATCCG
CCAAGTACAATTTTTTACTCTTCGAAGACAGAAAATTTGCTGACATTGGTAA
TACATGAGCAAATTAAAGCCTTCGAGCGTCCCAAAACCTTCTCAAGCAAGG
TTTTCAGTATAATGTTACATGCGTACACGCGTTTGTACAGAAAAAAAAGAA
AAATTTGAAATATAAATAACGTTCTTAATACTAACATAACTATTAAAAAAAAT
AAATAGGGACCTAGACTTCAGGTTGTCTAACTCCTTCCTTTTCGGTTAGAGC
GGATGTGGGAGGAGGGCGTGAATGTAAGCGTGACATAACTAATTACATGAG
TCCCATTCGCCACCCGAAGGTGTTGCCCAGCCGGCGCCAGCGAGGAGGCT
GGGACCATGCCGGCCAAAAGCACCGACTCGGTGCCACTTTTTCAAGTTGAT
AACGGACTAGCCTTATTTTAACTTGCTATTTCTAGCTCTAAAAC**CCA**CGCGT
ACCTAGGATCCAACG(lock1)[or GGGTGCGTCGTGGTACATAA(lock2) or CCT
GGACTCGGCACCTTGAC(lock3) or GAGCGAAGGACGTGAATGGG(lock4)]GA
CGAGCTTACTCGTTTCGTCCTCACGGACTCATCAGTAATACTGAATCTCTTT
TTCCATGGAGGGCACAGTTAAGCCGCTAAAGGCATTATCCGCCAAGTACAA
TTTTTTACTCTTCGAAGACAGAAAATTTGCTGACATTGGTAATACAGTCAAA
TTGCAGTACTCTGCGGGTGTATACAGAATAGCAGAATGGGCAGACATTACG
AATGCACACGGTGTGGTGGGCCCAGGTATTGTTAGCGGTTTGAAGCAGGC
GGCAGAAGAAGTAACAAAGGAACCTAGAGGCCTTTTGATGTTAGCAGAAT
TGTCATGCAAGGGCTCCCTATCTACTGGAGAATATACTAAGGGTACTGTTGA
CATTGCGAAGAGCGACAAAGATTTTGTTATCGGCTTTATTGCTCAAAGAGA
CATGGGTGGAAGAGATGAAGGTTACGATTGGTTGATTATGACACCCGGTGT
GGGTTTAGATGACAAGGGTGACGCATTGGGTCAACAGTATAGAACCGTGG
ATGATGTGGTgTCTACAGGATCTGACATTATTATTGTTGGAAGAGGACTATTT
GCAAAGGGAAGGGATGCTAAGGTAGAGGGTGAACGTTACAGAAAAGCAG
GCTGGGAAGCATATTTGAGAAGATGCGGCCAGCAAAACTAAAAAACTGTAT
TATAAGTAAATGCATGTATACTAAACTCACAAATTAGAGCTTCAATTTAATTA
TATCAGTTATTACCCTATGCGGTGTGAAATA

Double underline indicates the *ura3*-N and the *ura3*-C. The bold section is PAM sequences. The underlined sections indicate the lock1 gRNA targeting sequence, the brackets are lock2, lock3, lock4 gRNA targeting sequence in order.

Nucleotide sequences of lock_*leu2* module

AACTGTGGGAATACTCAGGTATCGTAAGATGCAAGAGTTCGAATCTCTTAG
CAACCATTATTTTTTTCCTCAACATAACGAGAACACACAGGGGCGCTATCGC
ACAGAATCAAATTCGATGACTGGAAATTTTTTGTTAATTTCAGAGGTCGCCT
GACGCATATACCTTTTTCAACTGAAAAATTGGGAGAAAAAGGAAAGGTGA
GAGGCCGGAACCGGCTTTTCATATAGAATAGAGAAGCGTTCATGACTAAAT
GCTTGCATCACAATACTTGAAGTTGACAATATTATTTAAGGACCTATTGTTTT
TTCCAATAGGTGGTTAGCAATCGTCTTACTTTCTAACTTTTCTTACCTTTTAC
ATTTCAGCAATATATATATATATTTCAAGGATATACCATTCTAATGTCTGCCCC
TATGTCTGCCCCTAAGAAGATCGTCGTTTTGCCAGGTGACCACGTTGGTCA
AGAAATCACAGCCGAAGCCATTAAGGTTCTTAAAGCTATTTCTGATGTTCGT
TCCAATGTCAAGTTCGATTTCGAAAATCATTTAATTGGTGGTGCTGCTATCG
ATGCTACAGGTGTCCCACTTCCAGATGAGGCGCTGGAAGCCTCCAAGAAG
GTTGATGCCGTTTTGTTAGGTGCTGTGGGTGGTCCTAAATGGGGTACCGGT
AGTGTTAGACCTGAACAAGGTTTACTAAAATGAC**CCA**CGCGTACCTAGGAT
CCAACG(lock1)[or GGGTGCGTCGTGGTACATAA(lock2) or CCTGGACTCGGC
ACCTTGAC(lock3) or GAGCGAAGGACGTGAATGGG(lock4)]GCTGGAAGCCT
CCAAGAAGGTTGATGCCGTTTTGTTAGGTGCTGTGGGTGGTCCTAAATGGG
GTACCGGTAGTGTTAGACCTGAACAAGGTTTACTAAAAATCCGTAAAGAAC
TTCAATTGTACGCCAACTTAAGACCATGTAACTTTGCATCCGACTCTCTTTT
AGACTTATCTCCAATCAAGCCACAATTTGCTAAAGGTACTGACTTCGTTGTT
GTCAGAGAATTAGTGGGAGGTATTTACTTTGGTAAGAGAAAGGAAGACGAT
GGTGATGGTGTCGCTTGGGATAGTGAACAATACACCGTTCCAGAAGTGCAA
AGAATCACAAGAATGGCCGCTTTCATGGCCCTACAACATGAGCCACCATTG
CCTATTTGGTCCTTGGATAAAGCTAATGTTTTGGCCTCTTCAAGATTATGGA
GAAAAACTGTGGAGGAAACCATCAAGAACGAATTCCCTACATTGAAGGTT
CAACATCAATTGATTGATTCTGCCGCCATGATCCTAGTTAAGAACCCAACCC
ACCTAAATGGTATTATAATCACCAGCAACATGTTTGGTGATATCATCTCCGAT
GAAGCCTCCGTTATCCCAGGTTCCTTGGGTTTGTTGCCATCTGCGTCCTTGG
CCTCTTTGCCAGACAAGAACACCGCATTTGGTTTGTACGAACCATGCCACG
GTTCTGCTCCAGATTTGCCAAAGAATAAGGTTGACCCTATCGCCACTATCTT
GTCTGCTGCAATGATGTTGAAATTGTCATTGAACTTGCCTGAAGAAGGTAA
GGCCATTGAAGATGCAGTTAAAAAGGTTTTGGATGCAGGTATCAGAACTGG
TGATTTAGGTGGTTCCAACAGTACCACCGAAGTCGGTGATGCTGTCGCCGA
AGAAGTTAAGAAAATCCTTGCTTAAAAAGATTCTCTTTTTTTATGATATTTGT
ACATAAACTTTATAAATGAAATTCATAATAGAAACGACACGAAATTACAAAA
TGGAATATGTTCATAGGGTAGACGAAACTATATACGCAATCTACATACATTTA
TCAAGAAGGAGAAAAAGGAGGATAGTAAAGGAATACAGGTAAGCAAATTG
ATACTAATGGCTCAACGTGATAAGGAAAAAGAATTGCACTTTAACATTAATA
TTGACAAGGAGGAGGGCACCACACAAAAAGTTAGGTGTAACAGAAAATC
ATGAAACTACGATTCCTAATTTGATATTGGAGGATTTTCTCTAAAAAAAAAA
AAATACAACAAATAAAAAACACTCAATGACCTGACCATTTGATGGAGTTTA
AGTCAATACCTTCTTG

Double underline indicates the *leu2*-N and the *leu2*-C. The bold section is PAM sequences. The underlined sections indicate the lock1 gRNA targeting sequence, the brackets are lock2, lock3, lock4 gRNA targeting sequence in order.

Nucleotide sequences of “key” plasmid

gRNA expression cassette

GTATATGTGTTATGTAGTATACTCTTTCTTCAACAATTAAATACTCTCGGTAG
CCAAGTTGGTTTAAGGCGCAAGACTGTAATTTATCACTACGAAATCTTGAG
ATCGGGCGTTCGACTCGCCCCCGGGAGA**GATGGCCGGCATGGTCCCAGC**
CTCCTCGCTGGCGCCGGCTGGGCAACACCTTCGGGTGGCGAATGGGA
CTTACGTTGGATCCTAGGTACGCG(key1)[or TTATGTACCACGACGCACCC(k
ey2) or GTCAAGGTGCCGAGTCCAGG(key3) or CCCATTCACGTCCTTCGCTC
(key4)]GTTT*TAGAGCTAGAAATAGCAAGTTAAAATAAGGCTAGTCCGTTATCAACT*
*TGAAAAAGTGGCACCGAGTCGGTGCTTTT*TTTATTTTTTGTCACTATTGTTATG
TAAAATGCCACCTCTGACAGTATGGAACGCAAACTTCTGTCTAGTGGATAA
CAGAATTTTTCTATGGCCAATTTATTCTTTTTTTTTTCCCTTTTCTTAATGTGC
GTATCATTGCCGGGTTTGAAATTTCGAAATTAACTACTAAAAATTACCGCTT
TAATGTATGGAATATGTG

Double underline indicates the small nucleolar RNA 52 (*SNR52*) polymerase III promoter and wavy underline indicates the Suppressor 4 (*SUP4*) terminator. The bold is the hepatitis delta virus (*HDV*) ribozyme. The underlined section sections indicate the key1 gRNA sequence, the brackets are key2, key3, key4 in order. Italics indicate the structural RNAs.

2μ-targeted gRNA expression cassette

CCTGTTGTAATCGAGCTCGGAAGACTCTCCTCCGTGCGTCCTCGTCTTCAC
CGGTCGCGTTCCTGAAACGCAGATGTGCCTCGCGCCGCACTGCTCCGAAC
AATAAAGATTCTACAATACTAGCTTTTATGGTTATGAAGAGGAAAAATTGGC
AGTAACCTGGCCCCACAAACCTTCAAATGAACGAATCAAATTAACAACCAT
AGGATGATAATGCGATTAGTTTTTTAGCCTTATTTCTGGGGTAATTAATCAGC
GAAGCGATGATTTTTGATCTATTAACAGATATATAAATGCAAAAACTGCATA
ACCACTTTAACTAATACTTTCAACATTTTCGGTTTGTATTACTTCTTATTCAA
ATGTAATAAAAGTATCAACAAAAAATTGTTAATATACCTCTATACTTTAACGT
CAAGGAGAAAAAACCCCGGATTCTAGAACTAGTGGATCCCCCCCGGACGA
CAGAGAATTCAGTATTA**CTGATGAGTCCGTGAGGACGAAACGAGTAAGC**
TCGTCGAAGCATACGATACCCCGCA*GTTTTAGAGCTAGAAATAGCAAGTTAAA*
*ATAAGGCTAGTCCGTTATCAACTTGAAAAAGTGGCACCGAGTCGGTGCTTTT***GG**

**CCGGCATGGTCCCAGCCTCCTCGCTGGCGCCGGCTGGGCAACACCTT
CGGGTGGCGAATGGGAC**TCATGTAATTAGTTATGTCACGCTTACATTCACG
CCCTCCTCCCACATCCGCTCTAACCGAAAAGGAAGGAGTTAGACAACCTG
AAGTCTAGGTCCCTATTTATTTTTTTAATAGTTATGTTAGTATTAAGAACGTT
ATTTATATTTCAAATTTTTCTTTTTTTTCTGTACAAACGCGTGTACGCATGTA
ACATTATACTGAAAACCTTGCTTGAGAAGGTTTTGGGACGCTCGAAGGCTT
TAATTTGC

Double underline indicates the pGalS promoter and wavy underline indicates the *CYC1* terminator. The bold section is the hammerhead (HH) and HDV ribozyme. The underlined sections indicate the 2μ-targeted gRNA sequence. Italics indicate the structural RNAs.

The sequences of DNA information

"lunyu.txt" DNA sequences

TACCAGTACGTTATGCCTATAAACCACGCTGACGAATGTTCTTGGTACCGAG
TTGGGAGCTcatcctcacacagacgcttaacaggatcccaactgtgacatcgaacgctaacgacacgctaaacctg
aagaggaccttgaacgagacggtaacactgacaccaactggaacgactacaagcacacgtagacagaagaggaacgtc
agacctaagtcgaacctgactcagactgtgaacctgatggacagagctattccgatcctcagacctaatccgacactgacg
gttaatggcaacaggaaggtgaaccctaagtcgaacgcaatcctgatcggtactgtgatctgcactacgaacctcactgtca
cgctaacgtgtactcacacggatgcacctgagtggtaacctcagtggaaagcctaacggaagcatcacacctaacgacac
actgaacgcaaactgcagtcacataccgacacgaacgctaacacgaactcgaactgtgacgagtacttccaactgcagtg
caaacgcaagcatcagtgacaagtgcaacgacacacgaaacccaaatcgcaactccaatccgagtcctaacaggatcgt
gacctacaagtggaccatcagcacaactcacaagtgcacgcaaaggcttacactcacggttaacaggattcgcacatcca
accagacttggattcgcacgaacaatggcagtgagacctgaagttccatgtcgaacgagaacaccaacccaacaccaata
gcgaagagcagttccaagcgttaccctaagctcaacctgaacgcttaccctaggcttagggtaacgagtatacggaacgtg
aacgacatcaccaatcggatgggtaacggtactgtgaagcgtacgctaaccacaacttcgactgtgacaggtaagtcgac
gctaaagcgaacagtcaagcacacatcgaactgctagagcagaaccaacaggatcggtatcctcaaggtcaagggaatc
ctgacgctaaaggcaaagggaatcggtacgctaacccaatagccaacgctaacagcaacaggtaagcagacgctaactg
tgaagtccaagtgcaactgcaccagtacactgaagccaaagggtacgctaaagggaaagcacatcccaacaaccttacg
cagggaaatcagcttagccacccttacccttacatggaccatcacggatgcacggtactaccacagagacgctaactgcta
ctcgtaaggtgaagggtttgcctattccgttggagaccaacaaggtgacgaacacggaataccgttacgagacgctaacct
acactgtgacactgaagcgtaactgcaccagaacacagacacctatcccaacgctaattcgcatgtccacctgtaacgaca
tcccaactcacttgtcgacggttagaacgagtcacaacctgacgtagaagggaaacctgatccagacgctaatcccatcttg
gattggcaactgcaaccgaatgtcgtagcctacaaggatagcgaacctgatctggatcgcaagcatcacgatcaagggaa
tacgcatgtggatggcattgtcgtatggcttagccatggagactgacacgagaacgactacgctaacagtcatgaggatga
ccatagcctctacctagagcactcctacgttcaagaccatcctcaccctattagccatctccatcctcactggtaagaccaca
cacacgtacaacgcaacgctaatgtccacgttgaaggtgatggctacgctaacagtgaacgacaacccaacgctaatggt
gaacccatcggaaacggtaaactcctcgaagtcgacaatcctcacagctaactcgaccaagaactcgatgtgcaactcga
aggacaactcgacctagaagcacatgagcaacgacaactgctcgactaacgactcgctttcgttgaaggtgacctcaatcg
gaaacgagaagtcgtgaggttgaagcaggcttagtccattagccactccaaacgacacttcgacgctaattgccaaccta

ccgtaaactgcaaggagactagcaacgacacgagtacgctaacaacgaaccctactgagacgctaatcgacatcaggac
ttgctgagcaacactgatggtcaacctgaccagtacgctaactcagattgccactgtgattgccagacagaagcacactcca
accacaagacacaagaggaacctgataggcaccagtagcctttgtcagtctagcaacaggaagcgtacgacaaagacca
accgttcacacataggcatcacgagccttatgtgccaaccaactccaacgctaacgactactgcaacgaagagccttattgc
gaacctgatctcgaaggtgaagtccacgctaaagaggataggcatccgacaatggagggaattagccagtcacacgtcaa
caaggacagtgacgtctacgctaatcgtccatgagatccctacgctaatgggaaagacctagctgatggagaagaccacct
acacgctaatcgagacactgacgagtactgtgacagacacaggaaactgcataggcacctgtatctgcatcccaaacgca
atcgcttctacgtcgatcagaacgaccgaaactgtgacaaggacctctacgctaatccgtcagagtacgtgaacgctaaag
ctgatctggtgaaggcaacagataggcagccttacacctaccactcagagaaacgtccagttcagtggaactccaactgtc
caatggtgatcgagaacgaactggaccttcaaccacaagacgacgcttaacacgatctggaaggctatctggactctcacg
ctaatctggactaggatctggactctgaacagcacgctaaaggtgacaacgaacgacacctgtaacccacagctattagcc
accgtttcgttgatccacataggcaacccaacctacctactgaaccacatcgctactcacacgctaattcggacctcaatgac
gactgtgactcagacacgtactgtcttgtcggaattCCAACACCCAGAGAATTGCAGTGTGATCG
TGGGCGTGATTTCGAAGTACGTGTTGCT

"lzw.txt" DNA sequences

TACCAGTACGTTATGCCTATAAACCACGCTGACGAATGTTCTTGGTACCGAG
TTGGGAGCTcaatcgcaagtcgaagcctaagaggaaggcaaaggcaaatcggaagcctatacggaagggtaac
gtcaagcgtaagtcgaaggacaagtgcaagtggaaccgaaatcgcaatccgatctcgaagcacattgccattggcatcac
gatcaggatctcgaacaggaaggcaaagtggaagcctaagcacaactccaaccgaaatcggatccacaatccgaacagc
atgaggatgacgaacgtgaagtggaaggctaatcggaaggctaagctcaagtggaatcggatcaggaagcgaaaggag
aaggtgaagtggattcgcaagtggaagtgcaatcggatcgtcaaggtcaagggaaagtggatcagcaagtggatgacga
tgaggaacagcaatcgcatgacgatcgtcatcgcaaaccagaagctgaagcgtaaggagaagggaaaggctaagagca
caggaaagcacaaggacaatcggaactgcaaccgtaatcggaatggcaacacgaatggcacagagaatccgaatccga
agctgaatcggactcagaacctcatgacgaagggtaagctcaagctgaagcctatggcaaacgctaaggtgacactcatct
ggactcgtactgacatcctgactgagaaccgtactcagaccaagaagcgtatcgagatcgtgaagcacacagctacaggt
actacgactaggaagcgtacttcgaactccaaccacaacctgaccagtacctacaagtccacaacgaagaggaagccaa
cagagatgacgacctagaatcggactgagaacgacaatcggaacctcacgagtactcgtaagagcaagccaaagtggat
acgcactgtcacatggaccctaaaccagaagcgaaagaggaaggagaagagcaagcctaagctgaaggcaaaggcta
agaccacgactaagacgacttggagatggacagcaacaggaactaccactagcacttccaaggacaagaccaagctgaa
gacgacgtgaacgtgtagaacgagaaggaatcggaagcgtaaggacatggacacaggaagaggaaccccttagactcag
accaaagtgcaagctgaagtcgacggttactcgtacagtgacccttataggcagagacaagagcaagcgtaagggaaag
cgaagatcgacctgaacccaaacagcaagtgtgaagagcagtgctaagtcgaactccagtcacaatccgagcatcagac
agagactgagccaaagtggaaagaccagtctcatacgcagagcaactcagaggaacagcactactagcagctacaagc
gaagcgtaaacaccaatcggacaacccacaggaaaggctagctctagtgtcaggatcagcctaagcgaaagtcacaggct
aagggatgcagcgtagggttaatggcagaagcatacgcaatggcaactggaatcggaggagtagcgaaacctacaagg
gaaagcgtaagctgactagcacctgaagatcgaggaacaagcacacaggaaagtgcaacgcaaaggacaagcgtaag
cagacaagcaaggctaacggatacctcagggttaagctgtactggactcgttatccgaaggtgagggtaaacaggaatgg
ctaccagtacctgagacctacactgtacaggaatcggtagccatacccaacactgtagtcgaatccgtaggtctaccgaag
acacagtaccaccgttagtaggagttcgatcggtaagctgaactgctaggtctagcgaaagcgtagttggacctacaggaa
cagctgtagacgaagagacaaccagagactgaaggagacactgaagtgcaacaggtaggtctaggctaggttgattggc
agacctagacgtaaggctttcccaaaggagttccgaaagcgtttcgacttccactaacggaggacttaggtcaagacgtaa
gcgacctacactgagactcactaggagtagagcttctccacgtgatagtggtagcagaagctgtactccagctcaaggaac

ttacggagtagcacgaagaaggacaactcctaggctaggctaaagctgaccttcactgtcacctctacactgtaaccgacc
gaaagtacgacgaacacgatcacggttaggctaaatccgacgtagaagtggacgttgacgtctatagcgattccgattcgg
attgcgaagggtaacgagaagtggatccgtatctgcatccacatcctcatcccatcttgctctcacatctccaagtggtaacc
gatcggaatgacgaccttgacggaaactgctactggtaccaagaatcggaccacaaccagaacgtgaaaggctaaggtg
atacgcagcaactcttcgagcagtagctagagatcgtagcagaggaacacacagaagaggaaggctaagtggaaggtg
aagcgtaagaggtcctacaagagcagagacaaggtgagttcgaactcgaggaacttcaggacctgaatcggtaacaggtt
cacgaacctgaactcctcgtacagacactcgttcacactgaagacgtactccaacccttaggctaagtggataccgaagtc
gaagtggaaggagatggacaacggtaagaggaagcctacactcaacgtcaaggtgaaggtgaaggacaaggtgagca
actcaggatctaccttctggagacactccttgttcctcttgaccttccctttccgtaacaggtccttgaggtgataggctttgcct
actcagaacctgactcgaactcgttcacagacgcaatcactgaatcggacgagaagtcacatcaggtatcggacaggaac
actctgtccaaatccgagctgttagggtaggttcagacacagctgtagcctaaagaccttcgcaaagggtttcggaagaga
cagagtcaacccaaagacgagttggacgtcttggctttgggaatggcttgaattCCAACACCCAGAGAATT
GCAGTGTGATCGTGGGCGTGATTTCGAAGTACGTGTTGCT

“cry.bmp” DNA sequences

TACCAGTACGTTATGCCTATAAACCACGCTGACGAATGTTCTTGGTACCGAG
TTGGGAGCTcaatcgcaatcggaatggcaacaccaacacgaacagcaacaggaactccaatcgcaatccgaact
gcaatccgaaccagaacctcaacctgaactgcaactcgaaccgaaaccgtaacgacaacgagaacgtcaaccacaacct
gaacgctaacggaaacggaaacgtgaagacgaagagcaacgtgaacggtaagtcgaagtgcaacgctaagaggaagc
agaagctcaagtggaagccaaagcctaaccagaagctcaaggacaaccgtaagcgaaaggtgaagcctaaggagaag
ggaaatggcaaggcaatacgcaagccaaagggtaaggagatacgcaacgacaactggaaggcaatagcgaagagcaa
ggtgaaggacatcaccattgcgaaggtcaaggctattgcgaagcgaatcaggaactccatagccatccacataccgatcct
catcctgaatcggatccctatccgaatccgtaagtggatcgagatcgtgatcgagaacaggatcgctatcaggaagtgcatc
ggaatctccaacggtatgacgaagtcgatgaggattcgcatgtggaagggtatggtcatggacatggagatgtcgataggc
atgggtatggctaacgtcatggcaaagaggacatccacaagcaacgagacatggacaaggacaacgacacacaagtcc
acaccaacactgaagcacacacctacatcgaagaccaagacgacacgtacagtcacagacacagagacacagacagga
acaggtactaccaacctcacttcgaagcgtacttccaagcagactaggaacagcactctgactcagactagcactacgatc
cagacagcaaacacgaactggatccagatctgcaactcgactgcaactgagactgacactccaactgtcatcccaaccaa
caacaccacttgcaagggaacctagactgtgactcacatcggtaccactacctcaaccatcaacgcaacctctatcgtcacc
ttgactctcaacccaacctacacgaagacctgaaccctaaccgtaacgacaacgagtaccttgactcctatgtccacgacaa
ctctgactaggacaggaatgtggatgagcattggcacgctaacactcacgtagaccgaaagaaccacgactatcagcaca
gtgattgccaccaagacgtacagaagcagaaggatcgacagatcgatctcgactggtaccatcacgttgagacgaatcac
gagagtcagacacaccgttacctgtaccacaacgcttatggtgatgaccacgtcaaccgaaagaggtagtaccagttgcag
agtgacgttgacccaaacgttcacgagaagtctgagtctcacacgaagtacgagttccactcacagagagagagcaacga
acagaccaagacgtacgaagagtcacagaaccagtgagagacctagtggtagactgactgcaagcatcatacggaagct
gattccgagtgtcagactcagctacaagcgtagcactactcgtaagaccagccttagtcgaattcggagtcctagcacaacc
agaagacagagtgacacgtctagcatcacgatcagtaggagactgagttggacaaccaccgttacccttaccagtagcaa
caggttgacgactagggtaaccttcagaacgaccgtaaggagtacggttacagctactcgaaaccgaagtgcttatccgat
gtgcagggaatatggcatctgcagcagttacagcagtagctacacgtactcgtaaccgtactggtaccacagtcagagcgt
ttacctgtacccataagcgtaccgaagtggatacccttacgagacggatgcagcacaggtagtacgcaacttcgaggcaat
aacgctagaccacagctacagagtacggttagtcctatcgctagtgcaggatcaccttctagagcatggctacagcatagtg
gagcagatacgactaccagagtccaaggagaagctcaagcttgtacacctaggcataggcttacgctatacggaggtgtta
ccgtacctcttaggactagcagttagcgttcagcttcaggagaggattctcgaggaacttctccttacggacttggaggctatt

ccacagcacaagaggtagatccttagcctaacgcagtcgtaggttctacgtgttccgttagctgagccaaagttggttaggc
agagctttccactatcggaggcatgctgaggagtcgttactccagtgtcttgaccaggactttgaggtagagcaggctattgt
cctagaggtagtcgttcgcatagcctttgtggttggtcttcgctttgcgataggagttcccaagctgaatgggattggactagg
tgtcatggttgacgttctgctcaacgaccagaatcggattgggtttccagagcgaatcaccatacgagttggcataggtctac
ctgtcagcttcaacctcacgttcaggattgtcgttggcaactcgatcttccttgccttcagtctatccgtcaggtagccaatcac
agtatggctctctcttggagtctacgacacctttcggatcagagttcctgtctgagtctcgattgctgtctgacatgaggtctgtc
ttggtgttccgatacaggtcttgctctgtgtctgcaatcctgtacctcaggtgatctggatagggatcctctttcgtctccagtttc
ccatcctagtccagatccatctaagcctccctattcacctcctgttctacctcgacatactgcatctggtctccaaggaagttcg
agacttccttcctctcgtcttctccaacaggttcgtgaagctcttcagtgtcgctattgctctaccactcggtattgccatcggaa
ttcctcaacccttagcactcccttaacccatcgtgtatcagggcggccCCAACACCCAGAGAATTGCAG
TGTGATCGTGGGCGTGATTTCGAAGTACGTGTTGCT

“smile.bmp” DNA sequences

TACCAGTACGTTATGCCTATAAACCACGCTGACGAATGTTCTTGGTACCGAG
TTGGGAGCTaaatcgcaatcggaatggcaacaccaacacgaacagcaacaggaactccaacaccaatccgaact
ggaaccacaaccagaaccacaactcgaacccaaaccctaaccgaaaccgtaacgacaacaggaacctcaacgtgaacg
caaacctcaacgagaacggtaagaccaagacgaatcggaacgctaagtccaagtcgaacctgaagagcaagcacaagc
agaagtgcaagctgaagccaaagcagaagcgaaaccgtaagccaaaggagaagcctaagcgtaaggcaaactgcaag
gtcaagggtaagctgaaggctaagcgtataccgaaggacatagcgatacggaagacgaaggagaagcgaattgccattc
gcaacgacaaggtgatacggatcagcatcaccaactccaagtgcatctcgaagggaaagtcgatccacaacgtcaagtcc
atcctgatcccaaacgctatccgaaatggcatcctcatcgagaacagcatcgtgatccacatcggaatcgcaatccgtaacg
caatctcgatccctatgacgaaccgaatgtcgaaggctatggacatgtgcaacccaatgaccattgcgatgaggatggtgat
cacgatgggtatagccacaagcacaaccataggcaacgtgacaaggacacacacatcgaacggtatcgacaagcacac
atccacactgaacgagacacctaagaccacagtcacagacacaccaacacgtatgtggacaggtacagctacaacgaca
ctcactaggaaccagactagcaagctcacttccactaccaacggaacttggacacgaactacgatgagcaagtggactcc
aactcctacttgcactctcactgtgactgagactgtcactgctaactcgactcagactggaaagagcaccatcatcgctacca
gaaccacaacaggaactctgactcgtaactggacctagacacagaccaacatccagacccaaacctgaacagtgacctac
aatcgcaccgttaccgaaacagagaccttgaccctaactgacacgatcaccactaccagaacgaagatctgcacccttaag
gcaacgactacgacaacccaaactcgaacctcaacgtctattcggatggtcactgcaacgagtacgttgacgagaagaac
caccttcatctggacttcgacctctatcgtcacgtgtacgtcaattggcaccgtaagacgaaaccctactcagagaaggatac
cgacgagtagagagagagcaaatccgagaacgagtaccaagaggagaccaaccagtattccgagacagagagacatg
gcaactctgagaaggagtaggaccttgacagcaacatggactgctagtcgaaccaagagtctcagttccatgagcactcgt
acgtagagactcaacacgagtgcaacctcaagtccaagagctagtgtcagtagcacgtcaactgtcagtcagagatggac
ctctacgcttacgtacacggttaacggaagcatcagctgtagtggtagctctagccttagcgaaagacgtagatcgaggaa
gactggtagtgagaggactagcgtaaggagtaggtacaggatcaggttcaggttgacgcaaaggacaaggtgaaggtgt
aggtctaggctaagtacgagtctgagggtaagacctaggcaataacgcagaggaatggcttaagcgagtcgtagggaata
tcgcagcacataaccgtacaccacgaacagcctaaggaactacaggagactgtactcgacggtatactggagatccagca
ctagcaactacctgtaacggagactctacagctacaccacactcagctgaagtgtcatgacgtaccgaagctcaagcaact
acgtctaccacagctacatgtcctacctcagtcagaccaactagaggtacccttagtcgatggagtactgcagcacatagtc
ctagaccagggtttagccaactcactacgcttagcgttagctgtaggtgagcagttatggctaggtcagcagatagcacagc
agatagctctatccgagttggtagcgaagcaagagaccataggctttctcgtaagccacggaattctccagatccttaccgta
gctctactccagacacttacgcttacggtacgacttaccgttccgttagtggttcgagagtgagagtcacaggctttaggagt
agggttaggcaagaggtttctggttcggtttcgcattcgctttagcgttcaggagaggtacctgtttgctgtaggacttgcagtt

gccattgacgaggtagttggagttgtccttgcgaatctccatgggatagcgattgtggtaaggcttcgacacgtgatacagct
caaggtatcggagctcttagtgcttcctgtcacagaccacatcacagttcgtcacctagtcagagttggacttgaggacgtac
tcagtgttcccattggctactggatcaggtttccacttcctgacagactctaggagttcgtcttcgacaacctcttggtctcactc
tcagtacggttctctgtctccatctcctatcggtttccagaggtcattcggaatgtgctctgtgaggagatctgctatccgattca
gctagccttctcgttccaactccaagtcaacgtcacgatccagttcctacttgggtttccgattggtcttgcgttctgtctcatcgt
catggtccacatctcgaatacgcacacgattgctctccagatccgaaattgccttggagagtaccaagggttcagtcttgtgc
tcgttgtcagctacttggtcgtcaagagcaaggaagtcgtgttcgaacaggtgatcgctttcctgatcacgttcgtcataccgt
atcgcttcgatctctgagaagcgactcgaCCAACACCCAGAGAATTGCAGTGTGATCGTGGG
CGTGATTTCGAAGTACGTGTTGCT

“panda.bmp” DNA sequences

TACCAGTACGTTATGCCTATAAACCACGCTGACGAATGTTCTTGGTACCGAG
TTGGGAGCTaaatcgcaatcggaatggcaacaccaacaccaatccgaacaggaactccaacacgaactgcaact
ggaaccacaaccagaacctcaacctgaacccaaactccaaccgaaaccgaaacccaaacgagaacgtcaacgtgaacc
tcaaccgtaacggaaacaggaacgcaaagacgaagagcaacctgaacggtaagtcgaagaggaagtggaagcacaag
tcgaagtgcaagcacaagccaaacgcaaagctcaagctgaagcctaaggagaaccagaagcgtaagctcaatcggaat
ccgaatcgcaagcgtataccgaaggtcaagcgaaaggcaatacgcaagggtaactggaagggtaaccgtatacggatag
ccaaccacataggcattgcgaagtggaacgacaacgagattgccattcgcaacgtcatcagcaagaccatagccaactcg
atcacgaaggacaacgctaaggcaatcaccaacacgatccctaatggcatcagcaagtccatacgcatcgtcattcggatt
cgcatcctcatccacatccgaaacggaatgacgatcgacatgtccatgtcgatgagcatcaggatggacatggagatcctc
atcggtatcctgatcgtgatggctatggacatgtccacaacgatggtgatgaccacatcgacaaccaacgctaactgcatag
gcatgggaaagggaacacgtaaggtgacacagacagtcatgggaatcggaaaggtgacaaggacaggtacagacatc
gagatcacgactacgactagcaagaggatcgcaacacgaacactcacaaggaacagcatggtcatggtgatccagaacg
gtatggcaatgtggacttcgatcgctaaggctatccgtactgacaaggctacttgcatctcgattcggactcctaagctgact
gagatagcgacttccaccagaaccacaactccaacagagaccaagacctacaccaacacagctacctgaactcagatcgt
gaccttgatctggacccttaaccctacatggactgctaccgtaaccgttacgagaacgaagaccttcatctgcatggtcacct
ctacacctaccgaaatgaggacttggacacacaacgacaccaagatcgctaaccctacgtagacgttcactctgacgacaa
tcaggacggttaagggaagaacgacgcaaacgcaaactggtatgggtagatggatccgtactcgtacacgaagaagcag
accaactgtgaacgtgaagtgcactctgacaagcactgcaataccgatctcgacgatcacggaaattccgacctcaagaga
gaacagcagagtgagaagcacctcaaccctaagttggatcgcaagtacgatgagcagaccaacgaacacgagtagatcg
atcgagacatccagtcagagaaccagtccaagatccagtctcatgtgcagtggtagtcacagactcagtaggacgcttagt
ccaactctcacttccatggctagtgagaccactagagcaacccaaactccaagtcctagcttgatgaccagcaacacgaca
agcacaacatccaccagaaggaagactcgtagctgtagccaaatccagagtgcaagtgctaggtgaaggttcacggttag
agctagcaagacgtgaagactgagtggaaggaacagcgtaaggtagacaagcagggtaacgtacactcacagtgtgact
gtcacggtaacccaaagcctaacgtgtaagagcatgtcgtatccgtaagcgacactctactggagggttagaggaacgcta
actgcttacacctaccagtacctcaggtactactcgattgcgtacaggaggagaaggtcatacccaaggaacaagacgag
gtgttacggaagtgactacgagtaccactagtgcaagcctaggctttagtgcagcactacagacaggtagactccttagacc
agacactagcctacagtgtaggacacgttgtaggtcacttgcacgtcaacgacttagggaacgaacttaccgacgtctttac
ggtaggagaccatcactcgatagccttaggctaggcaaactcgatagcactaacgcttcacgagcgaattctggtacgctta
ggcaagttcgtagtggttcccataagccacagtgttcgagagctgattcgagttcggattcggtaggagtttgaccttgagctt
gagcttcagcttgaggttgtgcttacgcttgtgcttagcgttgtggtagtcgttcgcaatctccttgctgatccacttcggaaag
gtcttgtggtagtccttccgtactcacttgaggtatggcttcgacagagctaaggagttgacctaaccgttggcatagaggta
cgtgttggagtagcgattgtcgttcgtcttgggatcactctcactgtcaccatcaagctcacgattcggtacgagaagcagtta

gccattccgattcccaaccttgtaccgtttcctcttgctctacgactcaagctagacgtcaacgtctcacagttgctaggagag cgtatagacgttaggcacctagttctcgtcagactcagcttctaggagaccttctctgtctgcttctcgaacgagttctcgttctt cgtcttcctctcacagtcactccatcttccagtccactaggtgtagacgttcctactctggatcacctaggagtttctgcttacgc tccacatcacctaggaagtcctgaatgtggtccgtatctgcatctggttcacacttcctgtaacggtagcgaagcaagtcctct tctctcttcgcttcagtcatccgatctcagtccctttcgtcatctaggtagggaactggatctgtcttacggtcagtgttggcttga acgtctgactcaggttgatcctgatcgtctgcttgacactgaagctcgctttgactgtagagctcccatgcaccagctcgaC CAACACCCAGAGAATTGCAGTGTGATCGTGGGCGTGATTTCGAAGTACGTG TTGCT

Double underline indicates universal primer regions for sequencing and wavy underline indicates buffer regions.

References

[1] G. M. Church, Y. Gao, S. Kosuri, *Science* **2012**, *337* (6102), 1628, https://doi.org/10.1126/science.1226355.

[2] N. Goldman, P. Bertone, S. Chen, C. Dessimoz, E. M. LeProust, B. Sipos, E. Birney, *Nature* **2013**, *494* (7435), 77, https://doi.org/10.1038/nature11875.

[3] Y. Erlich, D. Zielinski, *Science* **2017**, *355* (6328), 950, https://doi.org/10.1126/science.aaj2038.

[4] Z. Ping, S. Chen, G. Zhou, X. Huang, S. J. Zhu, H. Zhang, H. H. Lee, Z. Lan, J. Cui, T. Chen, W. Zhang, H. Yang, X. Xu, G. M. Church, Y. Shen, *Nature Computational Science* **2022**, *2* (4), 234, https://doi.org/10.1038/s43588-022-00231-2.

[5] R. N. Grass, R. Heckel, M. Puddu, D. Paunescu, W. J. Stark, *Angew Chem Int Ed Engl* **2015**, *54* (8), 2552, https://doi.org/10.1002/anie.201411378.

[6] J. Bornhol, R. Lopez, D. M. Carmean, L. Ceze, G. Seelig, K. Strauss, *Acm Sigplan Notices* **2016**, *51* (4), 637, https://doi.org/10.1145/2872362.2872397.

[7] M. Blawat, K. Gaedke, I. Hütter, X. M. Chen, B. Turczyk, S. Inverso, B. W. Pruitt, G. M. Church, *Procedia Comput Sci* **2016**, *80*, 1011, https://doi.org/10.1016/j.procs.2016.05.398.

[8] S. Yazdi, R. Gabrys, O. Milenkovic, *Sci Rep* **2017**, *7* (1), 5011, https://doi.org/10.1038/s41598-017-05188-1.

[9] L. Organick, S. D. Ang, Y. J. Chen, R. Lopez, S. Yekhanin, K. Makarychev, M. Z. Racz, G. Kamath, P. Gopalan, B. Nguyen, C. N. Takahashi, S. Newman, H. Y. Parker, C. Rashtchian, K. Stewart, G. Gupta, R. Carlson, J. Mulligan, D. Carmean, G. Seelig, L. Ceze, K. Strauss, *Nat Biotechnol* **2018**, *36* (3), 242, https://doi.org/10.1038/nbt.4079.

[10] M. Dimopoulou, M. Antonini, P. Barbry, R. Appuswamy, *Eur Signal Pr Conf* **2019**, https://doi.org/10.23919/eusipco.2019.8902583.

[11] W. H. Press, J. A. Hawkins, S. K. Jones, Jr., J. M. Schaub, I. J. Finkelstein, *Proc Natl Acad Sci U S A* **2020**, *117* (31), 18489, https://doi.org/10.1073/pnas.2004821117.

[12] W. Chen, M. Han, J. Zhou, Q. Ge, P. Wang, X. Zhang, S. Zhu, L. Song, Y. Yuan, *Natl Sci Rev* **2021**, *8* (5), nwab028, https://doi.org/10.1093/nsr/nwab028.

[13] M. Lu, Y. Wang, W. Qiang, J. Cui, Y. Wang, X. Huang, J. Dai, *Sci China Life Sci* **2023**, *66* (6), 1447, https://doi.org/10.1007/s11427-022-2252-0.

[14] T. A. Welch, *Computer* **1984**, *17* (6), 8, https://doi.org/10.1109/mc.1984.1659158.

[15] A. P. Cuny, K. Tanuj Sapra, D. Martinez-Martin, G. Flaschner, J. D. Adams, S. Martin, C. Gerber, F. Rudolf, D. J. Muller, *Nat Commun* **2022**, *13* (1), 3483, https://doi.org/10.1038/s41467-022-30781-y.

[16] G. E. Moore, *Proceedings of the Ieee* **1998**, *86* (1), 82, https://doi.org/10.1109/Jproc.1998.658762.
